# The gut microbiota‐derived metabolite indole‐3‐propionic acid enhances leptin sensitivity by targeting STAT3 against diet‐induced obesity

**DOI:** 10.1002/ctm2.70053

**Published:** 2024-11-28

**Authors:** Zhiwei Wang, Shaying Yang, Liangju Liu, Aiqin Mao, Hao Kan, Fan Yu, Xin Ma, Lei Feng, Tingting Zhou

**Affiliations:** ^1^ Department of Pharmacology Wuxi School of Medicine Jiangnan University Wuxi China; ^2^ Medical Basic Research Innovation Center for Gut Microbiota and Chronic Diseases Wuxi School of Medicine Jiangnan University Wuxi China

**Keywords:** gut microbiome, IPA, leptin sensitivity, metabolism, obesity, STAT3

## Abstract

Obesity is associated with the gut microbiome. Here, we report that gut commensal *Clostridia* bacteria regulate host energy balance through the tryptophan‐derived metabolite indole‐3‐propionic acid (IPA). IPA acts as an endogenous leptin sensitiser to counteract obesity. Mechanistically, IPA is secreted from the gut into the circulation, and then targets to the STAT3 in the hypothalamic appetite regulation centre, promoting its phosphorylation and nuclear translocation, which enhances the body's response to leptin, and regulates the balance between appetite and energy metabolism. The in vitro pull‐down assays involving site‐directed mutagenesis demonstrate that Trp623 in the SH2 domain is the key binding site for STAT3‐IPA interaction. High‐fat diet (HFD), rather than genetic factors, induces excessive secretion of antimicrobial peptides by Paneth cells, inhibiting the growth of *Clostridia* in the gut and resulting in decreased production of the beneficial metabolite IPA. IPA or *Clostridium sporogenes* supplement effectively controls weight gain, improves glucose metabolism, and reduces inflammation in DIO mice. IPA fails to achieve such effects in ob/ob mice, while exogenous leptin administration restores the therapeutic effect of IPA. Our study suggests that the IPA‐based gut‐brain axis regulates host metabolism, and supplementation with microbiome‐derived IPA could be a promising intervention strategy for treating obesity.

## Graphical abstract

- IPA‐based gut‐brain axis regulates host metabolism.
- IPA is a leptin sensitiser.
- STAT3 protein is the target of IPA.
- The circulating levels of IPA are affected by a high‐fat diet.

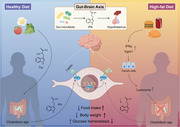

## 1 INTRODUCTION

The rising global rates of obesity and its related complications, including type 2 diabetes and hypertension, have become a major public health issue.^1^ Currently, ∼39% to 49% of the world's population, equivalent to 2.8 billion to 3.5 billion people, are affected by overweight or obesity.^2^, ^3^ Although, genetic factors also significantly impact an individual's susceptibility to developing obesity.^4^, ^5^ The balance between energy intake and expenditure is highly sensitive to external factors, including a sedentary lifestyle and consumption of high‐calorie foods intake, making environmental influences turn to be a key contributor to obesity.^6^

Leptin, a hormone originating from adipocytes, regulates food intake and energy expenditure by influencing the central appetite control centres in the brain, thereby maintaining weight balance.^7^ In individuals with normal body weight, leptin initially binds to leptin receptors (LepRb) on the surface of hypothalamic neurons, changing the LepRb conformation, which activates Janus kinase 2 (JAK2). Activated JAK2 phosphorylates multiple tyrosine residues on LepRb, which in turn recruits and activates signal transducer and activator of transcription 3 (STAT3). Phosphorylated STAT3 forms dimers and translocates to the nucleus, where it regulates the transcription of specific genes.^8^, ^9^ LepRb is also subject to inhibitory signals from several negative feedback mechanisms, including suppressor of cytokine signalling 3 (SOCS3) and protein tyrosine phosphatase 1B (PTP1B), ensuring that LepRb activation remains within physiological limits.^8^ The prevalence of leptin resistance in obese individuals limits its application in treatment for obesity.

Emerging researches have progressively pointed to the significant role of gut microbiota in the underlying mechanisms of obesity.^10^, ^11^, ^12^ The gut microbiota can directly interact with the host's intestinal epithelial cells and affect distant organs, including adipose tissue, liver, and central nervous system by synthesising and releasing metabolites into the circulation, thereby participating in the host metabolism.^13^, ^14^, ^15^ These pieces of evidence underscore the significant role of cross‐talk between gut microbial communities and their derived metabolic products with host organs in regulating organismal energy. If obesity arises as a consequence of dysbiosis in the gut microbiota–host symbiosis affecting energy metabolism, then understanding the potential communication mechanisms therein may aid in driving the development of antiobesity pharmaceuticals.

## 2 MATERIALS AND METHODS

Detailed methods are described in the Supplemental Materials and Methods.

### 2.1 Human subjects

The study received approval from the Ethics Committee of Jiangnan University, and informed consent was obtained from all participants. The research was conducted in accordance with the principles of the Helsinki Declaration. Fasting participants for targeted metabolomics research were recruited from individuals undergoing hospital examinations. The obese group (BMI > 28, *n* = 21) and the healthy group (BMI < 28, *n* = 21) were both of Han ethnicity. FMT donors were selected from these two cohorts. Individuals with systemic diseases (including liver diseases, gastrointestinal diseases, kidney diseases with serum creatinine levels > 2.0 mg/dL, autoimmune diseases, or malignancies) were excluded from this study. The individuals who had used antibiotics in the past three months were also excluded. General information, including age, gender, occupation, education level, smoking habits, and medication use (including antibiotic supplements), was obtained through standardised questionnaires. Baseline characteristics of the obese and healthy groups are provided in Table S1. Baseline characteristics of the FMT donors are provided in Table S2.

### 2.2 Mice

All animal experiments were conducted in full compliance with the protocol approved by the Animal Care Committee of Jiangnan University. Six‐week‐old male C57BL/6 mice were kept in a SPF environment. After a 1‐week acclimatisation period on a standard chow diet, the experiments were initiated.

In the HFD model, mice were fed a diet with 45% fat calories (TP23100, TROPHIC), while the control group received a chow diet with 11% fat calories. Food intake was recorded weekly throughout the experiment. At week 16, fat volume was evaluated utilising an in vivo optical imaging system (IVIS SPECTRUM, PerkinElmer, USA).

Male homozygous ob/ob mice (B6/JGpt‐Lepem1Cd25/Gpt) were utilised as a genetic obesity model (severely obese mice lacking leptin). These mice were purchased at 4 weeks of age (Gempharmatech Co., Ltd, China) and maintained under the same conditions as described above. They were fed a standard chow with 11% fat calories and randomly divided into IPA and Vehicle groups, each consisting of 6 mice.

### 2.3 Microorganisms

*Clostridium sporogenes* (ATCC^®^ 15579) was grown in TYG medium, which consisted of 3% pancreatic casein digest, 2% yeast extract, and .1% sodium thioglycolate. Inoculation and growth were performed under anaerobic conditions within a chamber filled with a gas mixture composed of approximately 80% nitrogen, 15% carbon dioxide, and 5% hydrogen. The cultivation was maintained at 37°C. OD600 was measured by BioTek Epoch2.

To evaluate the effect of freshly cultured *C. sporogenes* on DIO, antibiotic‐treated (Abx) mice were administered daily oral gavage of *C. sporogenes* suspended in sterile anaerobic PBS, at a dose of 1 × 10^8^ CFU per .2 mL, over a 10‐week period. The control group received an equivalent volume of PBS orally. Another group received treatment with heat‐killed (pasteurised) *C. sporogenes* (HFD + pasteurised *Cs*) by culturing in a casein yeast extract medium to confirm the absence of viable cells. Additionally, a group received simultaneous oral gavage of *C. sporogenes* and vancomycin (.5 g/L) in the drinking water to suppress the proliferation of *C. sporogenes* in the intestinal tract (HFD+Van+*Cs*).

### 2.4 FMT

The researchers collected and froze a fecal sample from each participant, storing them at ‐80°C. To colonise the gastrointestinal tract of antibiotic‐treated mice with human gut microbiota, frozen fecal samples were thawed in an anaerobic chamber. Equivalent portions from different donors within the same group were mixed and resuspended in prereduced PBS at a concentration of 100 mg/mL. The suspension was homogenised and filtered through a 70 µm mesh filter. The experimental mice were orally gavaged with 200 µL of the filtrate once a week.

### 2.5 Leptin sensitivity measurement

To evaluate the response to leptin administration, procedures outlined in a previous study were followed.^16^ Recombinant mouse leptin 116–130 aa (SCSLPQTSGLQKPES‐NH2, China Peptides Co., Ltd.) was dissolved in .9% sodium chloride solution and subsequently sterile‐filtered using a .22 µm filter. Mice were divided into two subgroups within each group, receiving either leptin (1 mg/kg) or vehicle, administered intraperitoneally twice daily for three consecutive days. At 90 min post the final injection, mice were euthanised, swiftly dissected, and the hypothalamus was rapidly frozen for immunoblotting analysis of phospho–STAT3 (Y705) and STAT3.

### 2.6 Re‐analysis of public metagenomic data

In this study, publicly available datasets from two obesity studies were downloaded from the European Nucleotide Archive (ENA) to analyse the abundance of *Clostridium* spp. The accession numbers for these datasets are PRJNA648796 (https://www.ebi.ac.uk/ena/browser/view/PRJNA648796) and PRJEB14215 (https://www.ebi.ac.uk/ena/browser/view/PRJEB14215).^17^, ^18^ The analysis included data from 137 obese individuals and 109 lean individuals. The downloaded data consisted of trimmed metagenomic sequencing reads, which were assembled using the MEGAHIT assembler. Open reading frame (ORF) prediction was performed using MetaGeneMark, and redundancy was removed using CD‐HIT. The abundance of each gene within the samples was determined by considering the number of aligned reads relative to the gene length. Sequence alignment was performed using the DIAMOND software. To calculate the abundance at a specific taxonomic level, the abundances of all genes associated with that taxon were summed.

### 2.7 Molecular docking

The protein crystal structures of STAT3 (PDB ID: 1BG1), PTP1B (PDB ID: 1NNY), SOCS3 (PDB ID: 2HMH), PTPN11 (PDB ID: 5EHR), LepRb (PDB ID: 8AVC), and JAK2 (PDB ID: 8C09) were downloaded from PDB. Molecular docking with IPA was performed using CDOCKER in Discovery Studio. To identify key amino acids within the active site, the Calculate Mutation Energy (Binding) tool was utilised to conduct virtual amino acid mutations based on interaction forces within the STAT3‐IPA complex.

### 2.8 STAT3 plasmid mutation

A mutant plasmid STAT3^W623A^ was constructed based on pCMV3‐Flag‐hSTAT3. The mutation primers were as follows: Forward: 5′ GGCGTCACTTTCACTGCCGTGGAGAAGGACATC 3′; Reverse: 5′ GATGTCCTTCTCCACGGCAGTGAAAGTGACGCC 3′. Amplification was carried out using KOD‐Plus‐Neo. Post‐PCR, 1 µL of FastDigest DpnI (Thermo Scientific) was added to the amplification product and incubated at 37°C for 2 h to digest the template strand. The mutated plasmid was then transformed into DH5α cells.

### 2.9 Pull‐down assays

Pipette 100 µL of streptavidin magnetic beads and wash them three times with PBS. Add 100 µL of Bio‐IPA, IPA, or Biotin to the beads and incubate at 4°C for 30 min. Use a magnetic rack to capture the beads, then add the protein extract to the bead complexes and gently agitate at 4°C for 24 h. Capture the beads using a magnetic rack and wash them three times with PBS. Add 28 µL of PBS and 7 µL of 5 × loading buffer to the beads and heat at 95°C for 10 min. Utilise a magnetic rack to separate the beads, then collect the supernatant for subsequent WB.

## 3 RESULTS

### 3.1 Untargeted metabolomic analysis revealed an association between indole‐3‐propionic acid and obesity

**FIGURE 1:**
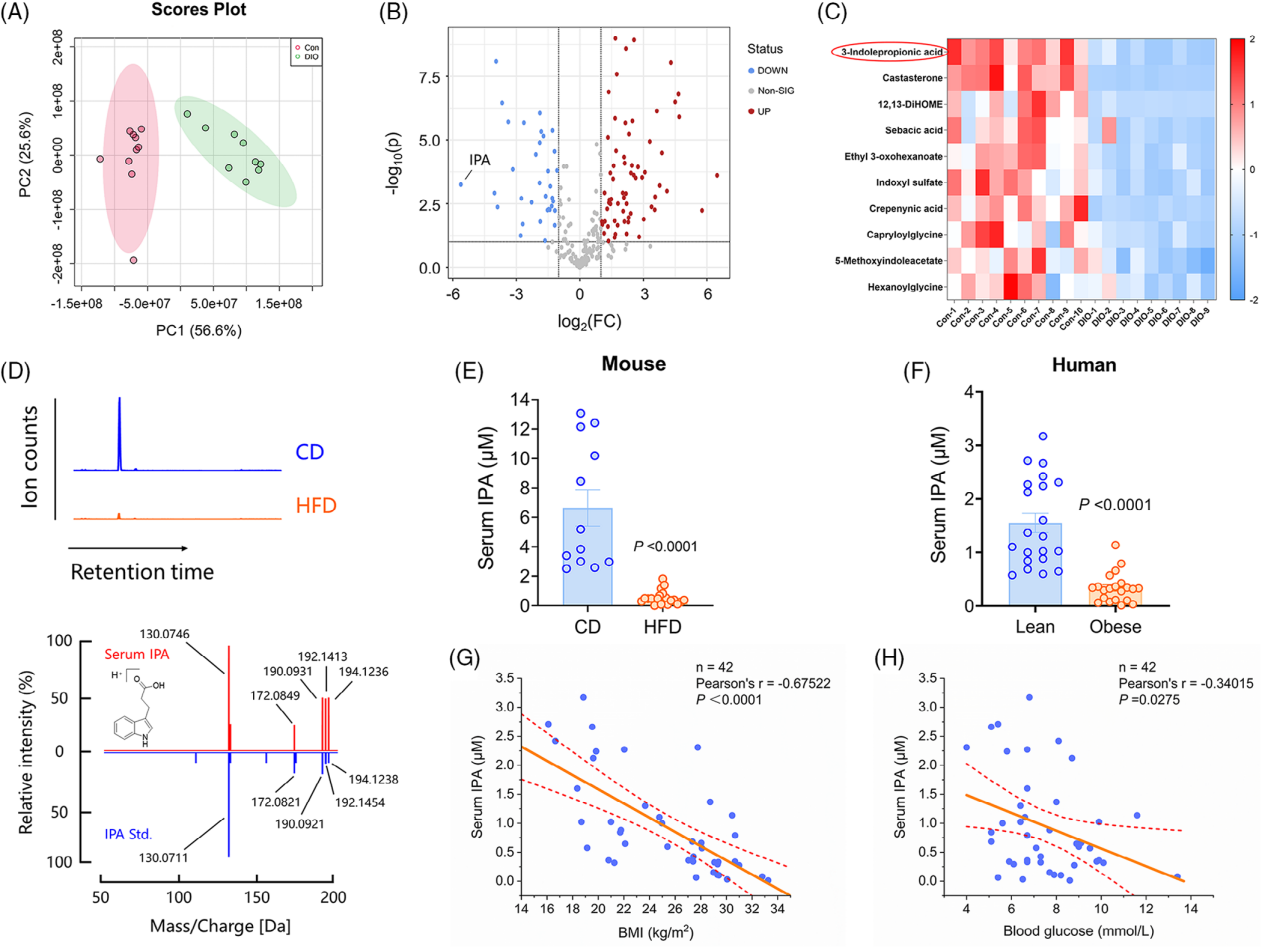
Untargeted metabolomics revealed an association between IPA and obesity. (A) Significant differences in serum metabolites between control (*n* = 10) and DIO (*n* = 9) mice as determined by principal component analysis. (B) Volcano plot illustrating the serum metabolomic differences between control and DIO mice. (C) Top 10 differentially decreased metabolites in the serum of DIO mice. (D) High‐resolution collision‐induced dissociation mass spectrum (red) of the serum metabolite *m*/*z* 190.0931 compared to synthetic IPA standard (blue). Extracted ion chromatogram peaks represent the relative ion counts of IPA. (E) Absolute quantification of IPA in the serum of CD mice (*n* = 12) and HFD mice (*n* = 20). (F) The levels of IPA in the serum of lean (*n* = 21) and obese (*n* = 21) individuals. (G) Pearson's correlation between IPA absolute concentration and body mass index. (H) Pearson's correlation between IPA absolute concentration and blood glucose. Data were presented as mean ± SEM. Statistical significance was determined by Mann–Whitney *U* test for E and F, and correlations were assessed using Pearson's correlation analysis for G and H.

We first performed untargeted metabolomics analysis on the serum of mice fed a chow diet and diet‐induced obese (DIO) mice. The findings indicated that high‐fat diet (HFD) led to metabolic disturbances in the mice (Figure 1A), affecting the abundance of numerous metabolites (Figure 1B). Here, we focused on the metabolites that were reduced in abundance under obese conditions. Upon listing the top 10 of these metabolites, we found that three of them were indole derivatives (Figure 1C). Metabolite Set Enrichment Analysis (MSEA) further indicated that the biosynthetic pathway of indole was impaired following HFD (Figure S1).

Indoles, being the deaminated products of tryptophan in both humans and mice, are microbially dependent and are undetectable in germ‐free mice.^19^ We focused on the top‐ranking substance (high‐resolution *m*/*z* 190.0931) led to its preliminary identification, based on monitored mass spectrometric features, as indole‐3‐propionic acid (IPA). Its origin primarily involves the metabolism of dietary tryptophan by *Clostridium* spp. in the host gut, subsequently leading to the direct secretion of IPA into the circulation.^20^

The untargeted metabolomic analysis, being a semi‐quantitative approach, necessitates validation and further quantitative analysis of candidate substances to confirm the observed associations. Utilising a UPLC system coupled with a Q‐TOF mass spectrometer, we definitively identified the serum metabolite with *m*/*z* 190.0931 as IPA. The analysed substance in serum exhibited identical high‐resolution MS/MS spectra and retention time as the authentically synthesised standard material (Figure 1D). The abundance of IPA in the serum of chow diet (CD) mice was approximately 2–14 µM, whereas HFD significantly reduced its concentration by about 12‐fold in obese mice (Figure 1E). Additionally, the serum levels of IPA were also significantly decreased in HFD‐induced obese female mice (Figure S2A and B). To examine whether IPA is associated with obesity‐related phenotypes, we measured serum IPA levels in 21 individuals with body mass index (BMI) greater than 28 and 21 individuals with BMI less than 28. The results indicated that the average IPA concentration in the serum of lean individuals was approximately 3.6 times higher than that of obese individuals (Figure 1F). Furthermore, Pearson's correlation analysis revealed a significant negative association between circulating IPA levels and both BMI and blood glucose levels (Figure 1G and H), suggesting that IPA may act as a molecular signal in the regulation of energy balance.

### 3.2 HFD aberrantly activates intestinal antimicrobial peptides, inhibiting the growth of *Clostridium* spp.

**FIGURE 2:**
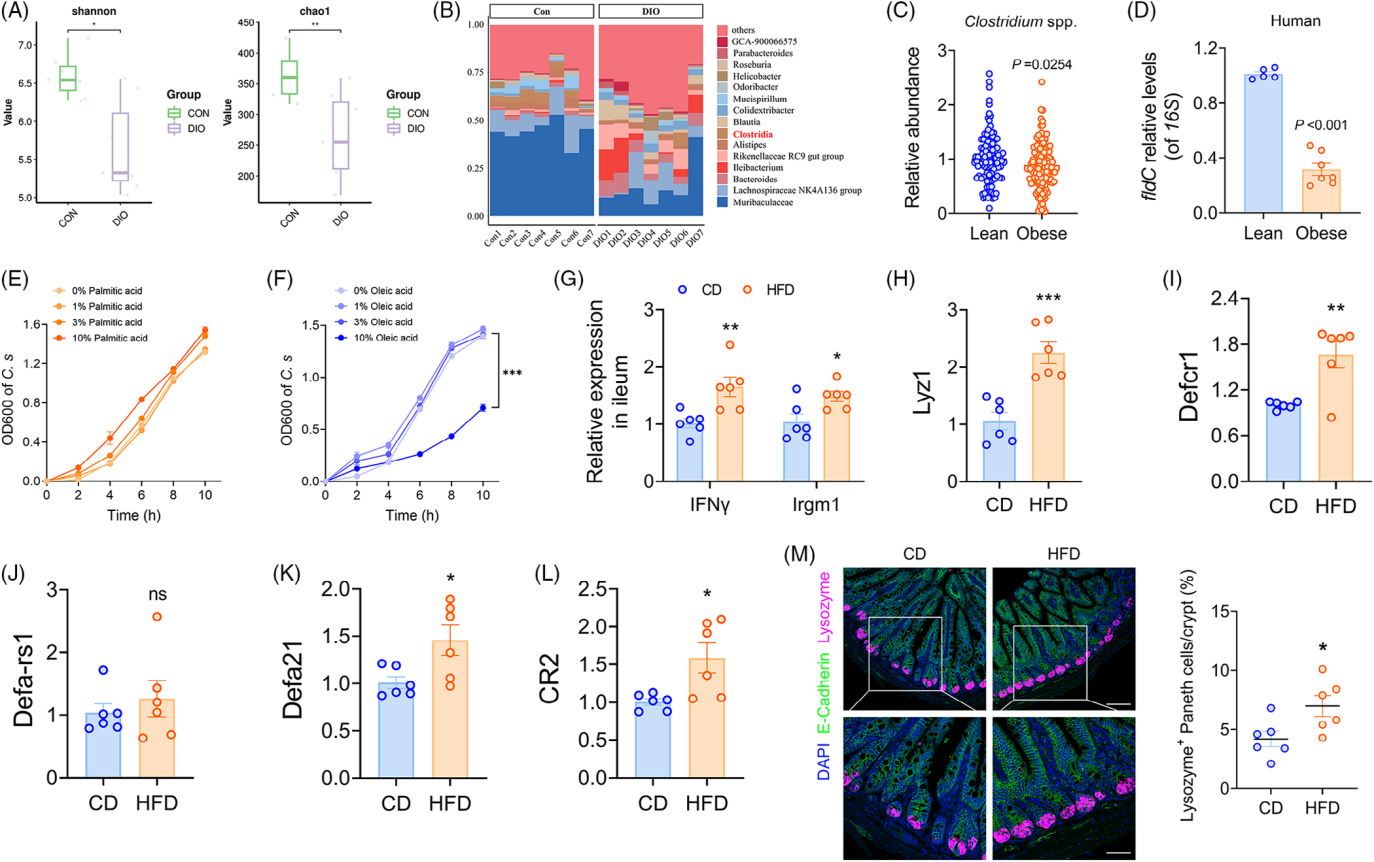
HFD aberrantly activates intestinal antimicrobial peptides, inhibiting the growth of *Clostridium* spp. (A) α‐diversity measures, including the Shannon index and Chao1 richness, of the gut microbiome in the two groups. (B) Genus‐level abundance of *Clostridia* in the feces of control (*n* = 7) and DIO mice (*n* = 7). (C) The prevalence and relative abundance of *Clostridium* spp. in human populations were analysed using publicly available metagenomic sequencing reads from the ENA database (PRJNA648796 + PRJEB14215), encompassing 137 obese and 109 lean individuals. (D) The levels of the *fldC* gene in fecal samples collected from lean (*n* = 5) and obese (*n* = 7) individuals. The growth curve of *C. sporogenes* exposed to different concentrations of palmitic acid (E) or oleic acid (F) in vitro. (G) *IFNγ* and *Irgm1*, (H) *Lyz1*, (I) *Defcr1*, (J) *Defa‐rs1*, (K) *Defa21*, and (L) *CR2* mRNA expression in the ileum of CD (*n* = 6) and HFD (*n* = 6) mice. (M) Quantification of lysozyme^+^ Paneth cells within mouse crypts (*n* = 6 mice per group), represented as the average number per crypt. Scale bars: 100 µm. Enlarged images are shown at the bottom. Scale bars: 50 µm. Data were presented as mean ± SEM. Statistical analysis was performed by Student's *t*‐test for A, D, G, H, I, J, K, L and M, by Mann–Whitney *U* test for C, and by two‐way ANOVA for E and F.

Given that IPA is a tryptophan metabolite derived from the gut microbiota, and that signals originating from the microbiota are known to regulate metabolism, with dysbiosis promoting obesity and related diseases, we performed 16S rRNA analysis on DIO mice and control mice. Consistent with previous studies, the composition of the gut microbiota in DIO mice showed significant differences compared to that of the control mice (Figure S3A). Compared to the control group, DIO mice exhibited lower bacterial diversity (Figure 2A), and the Bray–Curtis distance of the microbial community was higher in DIO mice (Figure S3B), indicating a more uneven community structure. Abundance differences in many species between the two groups also confirmed alterations in the gut microbiota, such as a relative decrease in *Muribaculaceae* and *Mucispirillum* in DIO mice, while *Blautia*, *Ileibacterium*, and *Rikenellaceae* *RC9 gut group* were enriched (Figure S3C). In particular, the genus level of *Clostridia*, capable of converting tryptophan into IPA, significantly decreased in DIO (Figure 2B). In comparison to the control group, we also identified functional alterations in the microbiome of DIO mice, encompassing various metabolic categories (Figure S3D). These changes included disruptions in the biosynthesis and metabolism of glycoproteins, amino acid metabolism pathways, and lipid metabolism pathways, suggesting a lower capacity for secondary metabolite biosynthesis in the gut microbiota of DIO mice.

We next investigated the prevalence and relative abundance of *Clostridium* spp. in human populations. We downloaded trimmed metagenomic sequencing reads from the European Nucleotide Archive (ENA) for healthy human subjects and morbidly obese patients (PRJNA648796 + PRJEB14215).^17^, ^18^ The re‐analysis included subjects from different regions worldwide, totalling 137 obese and 109 lean individuals. *Clostridium* spp. were detected in the feces of all human individuals, comprising .0057% to .49% of the total microbiota. In line with the findings observed in mice, the abundance of *Clostridium* spp. was significantly lower in the feces of obese patients compared to lean individuals (Figure 2C). The production of IPA relies on phenyllactyl‐CoA dehydratase β‐subunit, an enzyme encoded by the *fldC* gene.^21^ In the fecal samples we collected, obese individuals exhibited significantly lower levels of the *fldC* gene compared to lean controls (Figure 2D).

We hypothesise that gut Clostridia bacteria might be sensitive to HFD. To investigate which specific dietary component affects the growth of Clostridia bacteria, we measured the growth curves of *Clostridium sporogenes* exposed in vitro to varying concentrations of palmitic acid (a saturated fatty acid) or oleic acid (an unsaturated fatty acid). The growth curves showed no significant changes under different concentrations of palmitic or oleic acid (with only very high concentrations of oleic acid having an inhibitory effect), indicating that fatty acids at normal dietary concentrations do not affect the growth of *C. sporogenes* (Figure 2E and F).

Previous research has demonstrated that IFNγ stimulates Paneth cells to secrete antimicrobial peptides via the downstream signalling molecule Irgm1, affecting the colonisation and growth of gut microbiota.^22^ In this study, we observed that compared to CD, HFD significantly increased the expression of IFNγ and Irgm1 in the ileum (Figure 2G). Concurrently, the expression of several Paneth cell‐associated antimicrobial peptide genes, including *Lyz1* and members of the *Defa* family, sharply increased in the intestines of HFD mice (Figure 2H‐L). The excessive secretion of antimicrobial peptides likely contributes to the reduction in gut microbiota quantity and diversity in HFD mice. Histological analysis revealed a slight increase in the number of lysozyme‐expressing Paneth cells in the intestinal crypts of HFD mice compared to CD mice (Figure 2M). These findings suggest that HFD abnormally activates Paneth cells via the IFNγ‐Irgm1 axis, leading to the overproduction of antimicrobial peptides, which suppresses the growth of *Clostridia* bacteria and consequently reduces IPA synthesis.

### 3.3 The gut microbiota influences host metabolism

**FIGURE 3:**
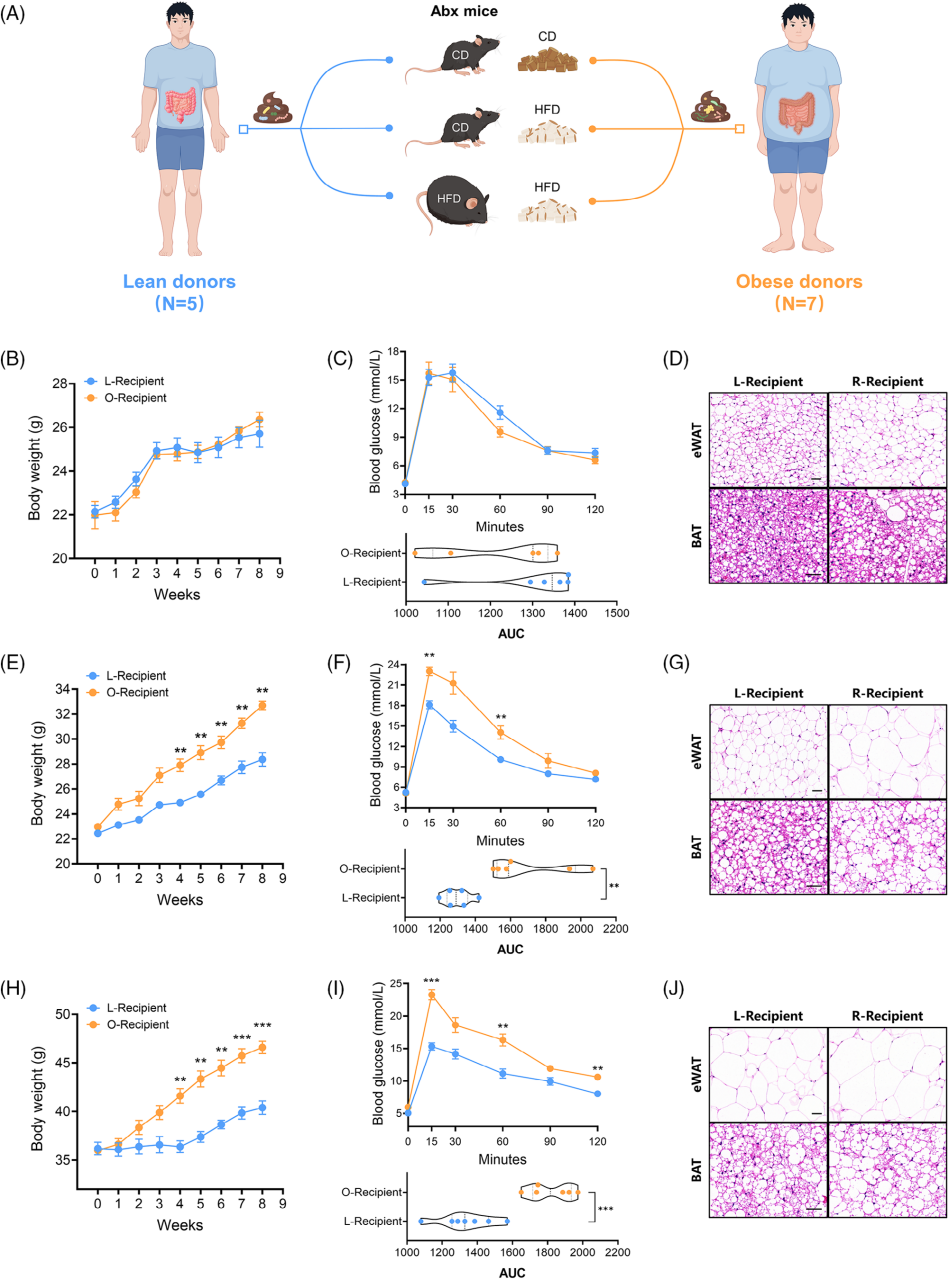
The gut microbiota influences host metabolism. (A) Experimental design schematic for FMT. Fecal microbiota from lean donors (*n* = 5) or obese donors (*n* = 7) was transplanted into three different groups of antibiotic‐treated mice. The first group of chow diet mice maintained a chow diet after FMT, with monitoring of weekly body weight (B), glucose tolerance at the end of FMT (C), and adipocyte morphology in eWAT and BAT (D). The second group of chow diet mice transitioned to an HFD after FMT, with monitoring of weekly body weight (E), glucose tolerance at the end of FMT (F), and adipocyte morphology in eWAT and BAT (G). The third group consisted of DIO mice maintaining an HFD after FMT, with monitoring of weekly body weight (H), glucose tolerance at the end of FMT (I), and adipocyte morphology in eWAT and BAT (J). Scale bars: 40 µm. Data were presented as mean ± SEM. For B, C, E, F, H and I, statistical analysis was performed by two‐way ANOVA. For AUC in C, F and I, statistical analysis was performed by Student's *t*‐test.

To determine whether the gut microbiota is sufficient to induce metabolic phenotype changes, we subjected mice to a 2‐week broad spectrum antibiotics cocktail treatment (Abx), followed by random allocation to receive fecal microbiota transplants (FMT) from either lean (BMI < 24, *n* = 5) or obese individuals (BMI > 30, *n* = 7) (Figure 3A). After fecal microbiota transplantation (FMT), FMT did not exert any effect on their body weight and blood glucose under chow diet (Figure 3B‐D). However, if mice were switched to a high‐fat diet post FMT, regardless of their previous dietary regimen, mice colonised with obesity‐associated microbiota exhibited a significantly increased rate of weight gain, impaired blood glucose control, and lipid degeneration (Figure 3E‐J). Surprisingly, in DIO mice colonised with microbiota derived from lean individuals, their weight gain was notably inhibited (Figure 3H), and there was a reversal of whitening in BAT (Figure 3J). This suggests that certain potentially beneficial gut bacteria can help improve host metabolism and resist diet‐induced obesity.

### 3.4 *Clostridium sporogenes* aids the host in resisting DIO

**FIGURE 4:**
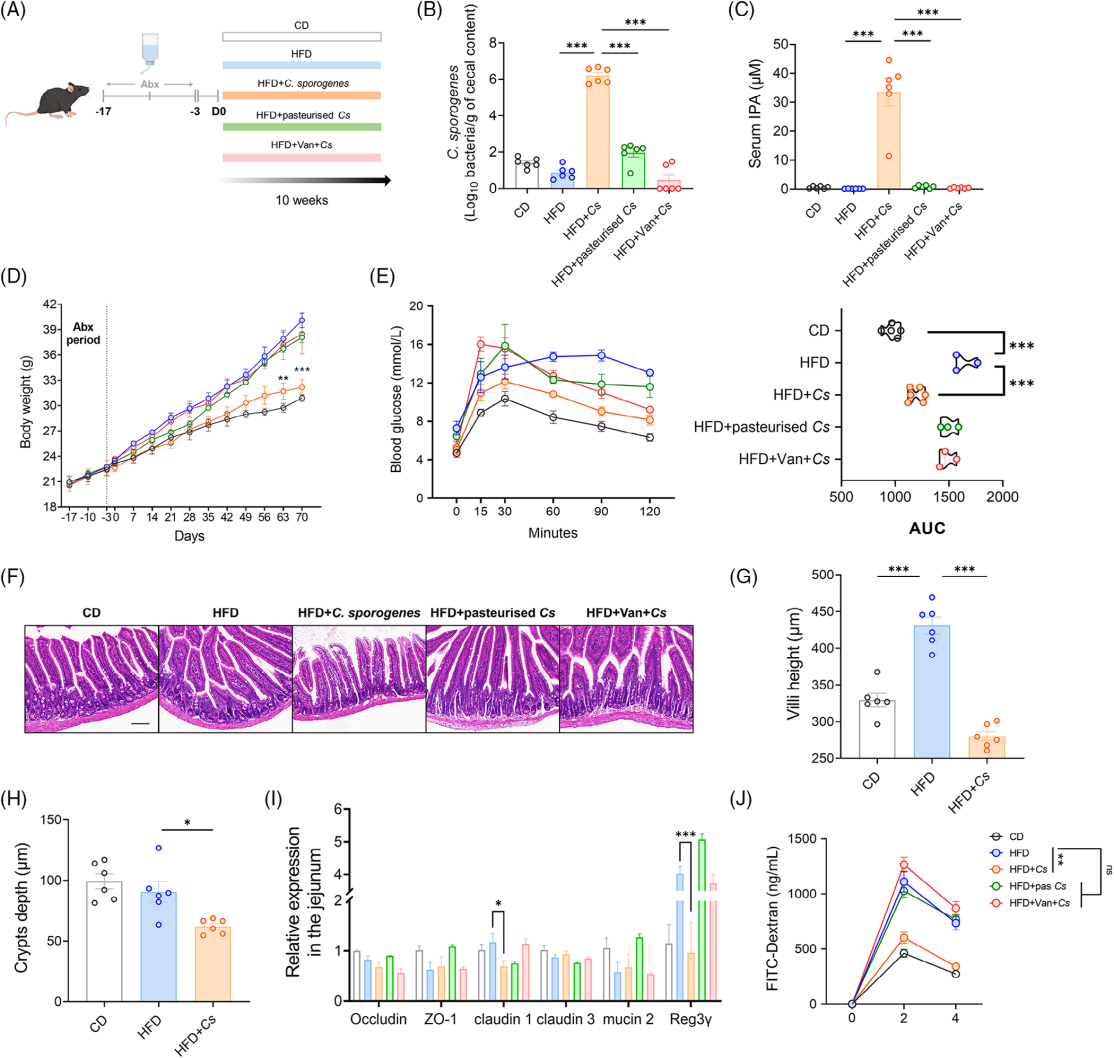
*Clostridium sporogenes* aids the host in resisting DIO. (A) Experimental design schematic. Mice were subjected to antibiotic pretreatment to clear native gut microbiota, followed by daily gavage of *C. sporogenes* (1 × 10^8^ cfu/200 µL), gavage of heat‐killed *C. sporogenes*, or addition of vancomycin (Van) to drinking water to reestablish gut microbiota. After 10 weeks of gavage, the abundance of *C. sporogenes* in the cecum (B) and the concentration of IPA in serum (C) were measured. (D) Weight gain curve. (E) A glucose tolerance test plotted with AUC. (F) Representative images of H&E staining in the jejunum. Scale bar: 200 µm. Height of jejunal villi (G) and depth of crypts (H). (I) Relative expression of genes related to intestinal barrier function in the jejunum. (J) Serum concentration of FITC‐dextran 2 and 4 h after oral gavage. Data were presented as mean ± SEM. Statistical analysis was performed using one‐way ANOVA for B, C, AUC in E, G, H and I, and two‐way ANOVA for D and J.

We have demonstrated the shift in gut microbial profiles in obesity and its impact on disease progression. However, the specific role of individual bacterial strains in the occurrence and progression of obesity remains insufficiently understood. Given that Indole derivatives are primarily synthesised from tryptophan by the Gram‐positive bacterium *Clostridium sporogenes*, we investigated the impact of *C. sporogenes* on obesity. Following a 14‐day treatment with a broad‐spectrum antibiotic cocktail to deplete the gut microbiota in mice, we replenished the intestinal tract of Abx‐treated mice with an in vitro cultured strain of *C. sporogenes*, under a high‐fat diet regimen (Figure 4A). After 10 weeks, a substantial colonisation of *C. sporogenes* was detected in the cecal contents of mice (Figure 4B). Alterations in the abundance of *C. sporogenes* in the gut led to a substantial rise in IPA levels in both serum and fecal samples (Figures 4C and S4A). *C. sporogenes*, in a mono‐colonised environment, could modulate the metabolic balance of mice, resulting in a 19.8% reduction in weight compared to the HFD group (i.e., 7.94 g) (Figure 4D), independent of the composition of the gut microbiota. This corresponded to a significant decrease in the mass of various adipose depots (i.e., subcutaneous, mesenteric, and epididymal) (Figure S4B). Furthermore, mice treated with *C. sporogenes* exhibited glucose clearance kinetics similar to CD mice, indicating that *C. sporogenes* could alleviate obesity‐induced insulin resistance (Figure 4E). Subsequently, we investigated whether the viability of *C. sporogenes* would affect its antiobesity effects. The use of pasteurisation or vancomycin treatment to kill *C. sporogenes* completely eliminated its beneficial effects on body weight and glucose homeostasis (Figure 4D and E).

HFD induction significantly led to an elongation of villi and deepening of crypts in the small intestine of Abx‐treated mice (Figure 4F). This results in a larger absorption area, slowing down the movement of chyme in the small intestine, aiding in the absorption of excess nutrients and exacerbating lipid absorption and accumulation. Live *C. sporogenes* reduced the height of the duodenal villi and the depth of the crypts (Figure 4G and H). The reduction in villi height might lead to a decrease in the absorption surface, reducing the organism's energy absorption. Notably, treatment with *C. sporogenes* reversed the abnormal elevations in *Occludin 1* and the antimicrobial defence protein *Reg3γ* induced by HFD, which is critical for maintaining intestinal barrier integrity (Figure 4I). In addition, the serum recovery rate of FITC‐dextran significantly decreased, indicating an improvement in the intestinal epithelial barrier (Figure 4J).

### 3.5 Supplementation with IPA alleviates DIO in mice

**FIGURE 5:**
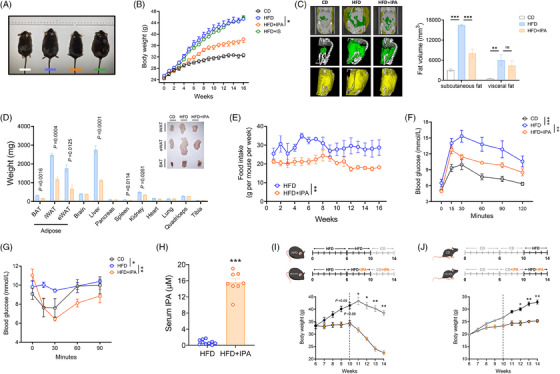
Supplementation with IPA can alleviate DIO in mice. (A) Comparison of body size among the groups of mice at the end of the treatment period. White: CD, chow diet (*n* = 6); Blue: HFD, high‐fat diet (*n* = 8); Orange: HFD+IPA (*n* = 8); Green: HFD+IS (*n* = 6). (B) Weight changes in mice fed an HFD and orally supplemented with IPA (20 mg/kg) or IS (20 mg/kg) daily for 16 weeks, as well as in mice fed CD or HFD with daily oral administration of the vehicle. (C) Three‐dimensional imaging and volume of mouse adipose tissue. Green: visceral fat; Yellow: subcutaneous fat. (D) Tissue weights and representative images of adipose tissue in HFD mice after 16 weeks of treatment with vehicle or IPA. BAT, brown adipose tissue; eWAT, epididymal white adipose tissue; iWAT, inguinal white adipose tissue. (E) Weekly food intake in HFD mice supplemented with IPA and HFD mice with vehicle supplementation. (F) Oral glucose tolerance test. (G) Insulin tolerance test. (H) Serum concentration of IPA in HFD mice after 16 weeks of vehicle or IPA treatment. (I, J) Experimental outline (above); weight response to IPA supplementation in mice on different diet regimens, with diet exchange at week 10. Data were presented as mean ± SEM. Statistical significance was determined by Student's *t*‐test for D and H, by one‐way ANOVA for C, and by two‐way ANOVA for B, E, F, G, I, and J.

To directly examine whether IPA supplementation affects the development of obesity, we administered HFD to 7‐week‐old mice, with daily oral gavage of IPA (20 mg/kg, *i.g. qd*) or vehicle (PBS) for 16 weeks. Additionally, we included a separate group of mice, which received another indole compound from the top10 list, indoxyl sulfate, via oral gavage. We observed a significant reduction in body size in IPA‐treated mice (Figure 5A), with effective weight control starting from the fourth week. After 16 weeks of HFD exposure, the IPA group exhibited a 31.07 ± 2.19% reduction in weight gain (Figure 5B). In contrast, indoxyl sulfate treatment showed no significant effect, indicating that not all indole compounds can combat obesity.

Three‐dimensional reconstruction of whole‐body adipose tissue using Micro‐CT revealed a marked reduction in visceral fat accumulation in IPA‐treated mice, with less pronounced effects on subcutaneous fat (Figure 5C). IPA‐treated mice also showed reduced mass of brown and white adipose tissues, liver, spleen, and kidneys, while the weights of other organs remained unchanged (Figure 5D). IPA treatment suppressed appetite in mice (Figure 5E). During the oral glucose tolerance test (OGTT), IPA‐treated mice demonstrated better glycaemic control, returning to baseline glucose levels more rapidly after peaking at 15 min post‐glucose administration (Figure 5F). Compared to HFD‐fed mice, IPA‐treated mice were more insulin sensitive (Figure 5G) and exhibited significantly improved lipid profiles (Figure S5A‐D). Furthermore, levels of resistin and glucose‐dependent insulinotropic polypeptide (GIP), which are excessively secreted under obese conditions, were significantly reduced, although adiponectin (ADPN) levels remained largely unchanged (Figure S5E‐G). These beneficial changes in mice were linked to elevated circulating levels of IPA (Figure 5H).

Building on these findings, we examined the impact of a 4‐week oral IPA administration on a new group of DIO mice following 6 weeks of HFD feeding. The results showed a significant reduction in body weight in the DIO mice (Figure 5I), while a more moderate effect was observed in lean mice maintained on a chow diet for 6 weeks (Figure 5J). Interestingly, similar outcomes were seen after switching diets at week 10 (Figure 5I and J). Overall, these results suggest that IPA treatment is effective in preventing both the onset and progression of DIO.

### 3.6 IPA alters the morphology and gene function of adipocytes

**FIGURE 6:**
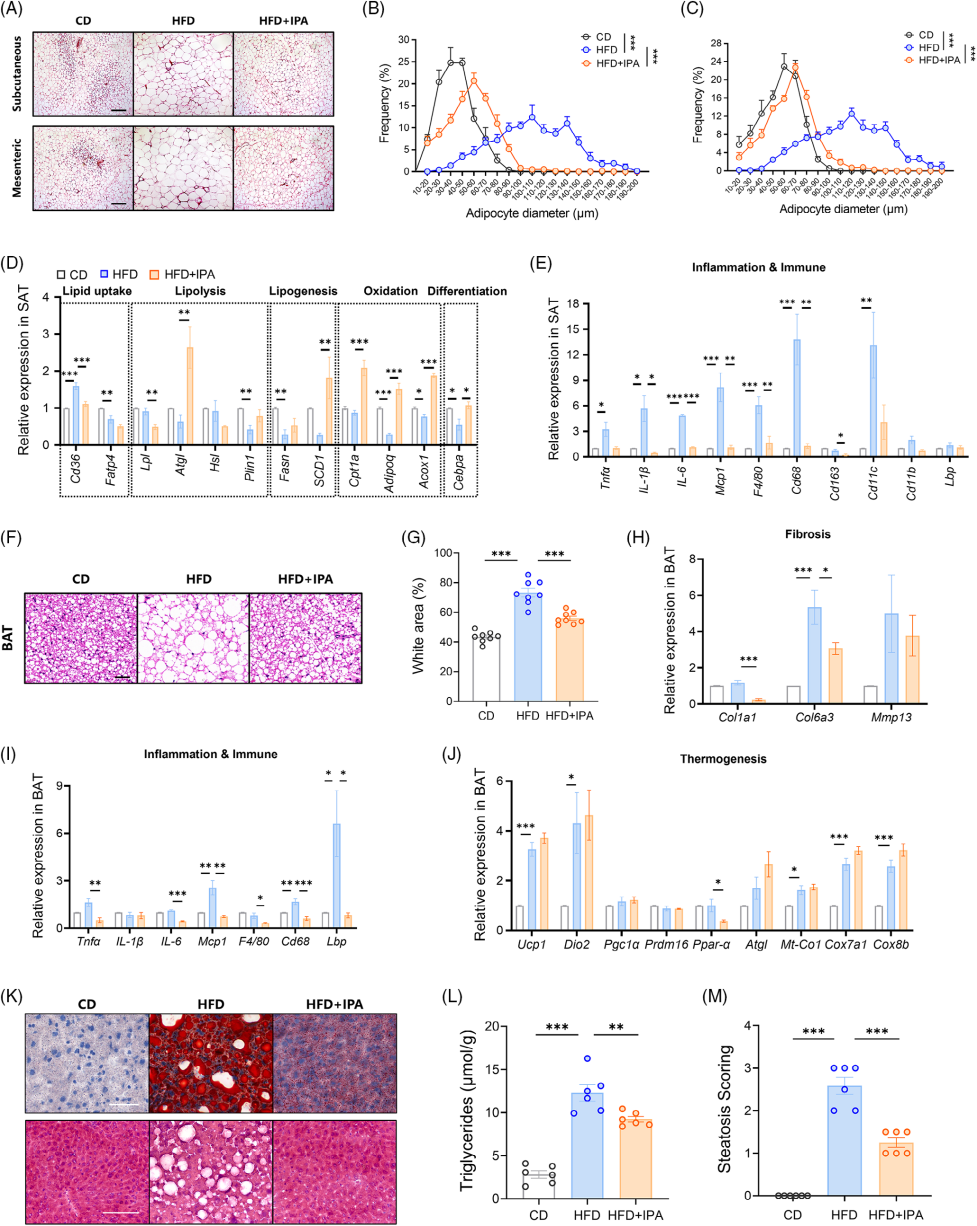
Impact of IPA on adipose tissue expansion, inflammation, and hepatic steatosis induced by HFD. (A) Representative images of H&E staining illustrating SAT and MAT. Scale bar: 200 µm. (B) Size distribution of adipocytes in SAT. (C) Size distribution of adipocytes in MAT. (D) Relative expression levels of genes associated with lipid metabolism in WAT. (E) Relative expression levels of genes linked to inflammation and the immune system in WAT. (F) Representative H&E staining images of BAT. The scale bar is 40 µm. (G) Percentage of white areas corresponding to lipid droplets in BAT slices. (H) Relative expression levels of genes related to the extracellular matrix and fibrosis in BAT. (I) Relative expression levels of genes associated with inflammation and the immune system in BAT. (J) Relative expression levels of genes related to thermogenesis in BAT. (K) Representative images of Oil Red O staining (above) and H&E staining (below) in the mouse liver. Scale bar: 100 µm. (L) Triglyceride content in the mouse liver. (M) Severity score of hepatic steatosis based on H&E results. Data were presented as mean ± SEM. Statistical significance was determined by two‐way ANOVA for B and C, and by one‐way ANOVA for D, E, G, H, I, J, L and M.

The pathogenesis of obesity and metabolic diseases is closely linked to the expansion, dysfunction, and inflammation of WAT.^23^ To assess adipocyte size, we performed histological analysis on both subcutaneous adipose tissue (SAT) and mesenteric adipose tissue (MAT) (Figure 6A). In both SAT and MAT, IPA treatment resulted in a notable increase in the proportion of small adipocytes and a decrease in the proportion of large adipocytes (Figure 6B and C). The expression of cluster of differentiation 36 (*CD36*), a white blood cell differentiation antigen involved in fatty acid uptake, exhibited a marked upregulation under HFD induction, which was restored to normal levels following IPA treatment. Additionally, treatment with IPA markedly increased the expression of genes involved in lipolysis and fatty acid mobilisation, including *Atgl* and *Plin1*. Moreover, there was a notable increase in the levels of markers for adipocyte differentiation and lipid oxidation were substantially increased after IPA treatment (Figure 6D).

Cytokines are essential regulators of adipocyte function,^24^ and it is well‐established that elevated levels of proinflammatory cytokines are linked to the onset of insulin resistance and obesity.^25^ The expression levels of a series of chemokines/cytokines, including *Tnf‐α*, *IL‐6*, *Mcp1*, *IL‐1β*, and macrophage infiltration markers (such as *F4/80*, *Cd68*, and *Cd11c*), were increased due to HFD, indicating active recruitment of macrophages into adipose tissue, a situation completely reversed by IPA treatment (Figure 6E).

HFD‐induced brown adipose tissue (BAT) enlargement is associated with an increase in lipid droplet volume (Figure 6F). The intracellular area occupied by lipid droplets significantly increased, and IPA restored the morphology of brown adipocytes and reduced lipid content (Figure 6G). HFD upregulated the expression of tissue necrosis factor‐related genes such as collagen and matrix metalloproteinases, while IPA normalised their expression (Figure 6H), indicating that IPA prevents HFD‐induced BAT remodelling and fibrosis. Additionally, IPA reduced inflammation (Figure 6I) and levels of *PPAR‐α* in BAT, linked to obesity and insulin resistance. However, IPA did not alter the expression of several key genes involved in the mitochondrial respiratory chain (Figure 6J).

### 3.7 IPA alleviates hepatic lipid accumulation in mice

Given the capacity of IPA to enhance mitochondrial fatty acid oxidation (Figure 6D), we investigated the impact of IPA treatment on hepatic lipid accumulation. Liver Oil Red O staining revealed a reduction in lipid deposition with IPA treatment (Figure 6K), and the hepatic triglyceride content, representing the storage form of excess lipids, also exhibited a decrease in mice (Figure 6L). Furthermore, IPA alleviated the progression of hepatic steatosis in these mice (Figure 6M).

### 3.8 The antiobesity effect of IPA depends on intact leptin signalling

Obesity is a metabolic disorder that arises from the complex interplay between genetic predispositions and environmental influences. We tested whether circulating levels of IPA are affected by genetically induced obesity (ob/ob). Unlike DIO, despite the spontaneous obesity observed in ob/ob mice (Figure S6A), their serum IPA concentrations were comparable to age‐matched wild‐type mice (Figure S6B). Furthermore, the levels of the *fldC* gene in the feces of ob/ob mice were not reduced (Figure S6C). This further confirms that gut *Clostridia* bacteria and their derived metabolite IPA are primarily influenced by dietary patterns rather than the host genetic background.

**FIGURE 7:**
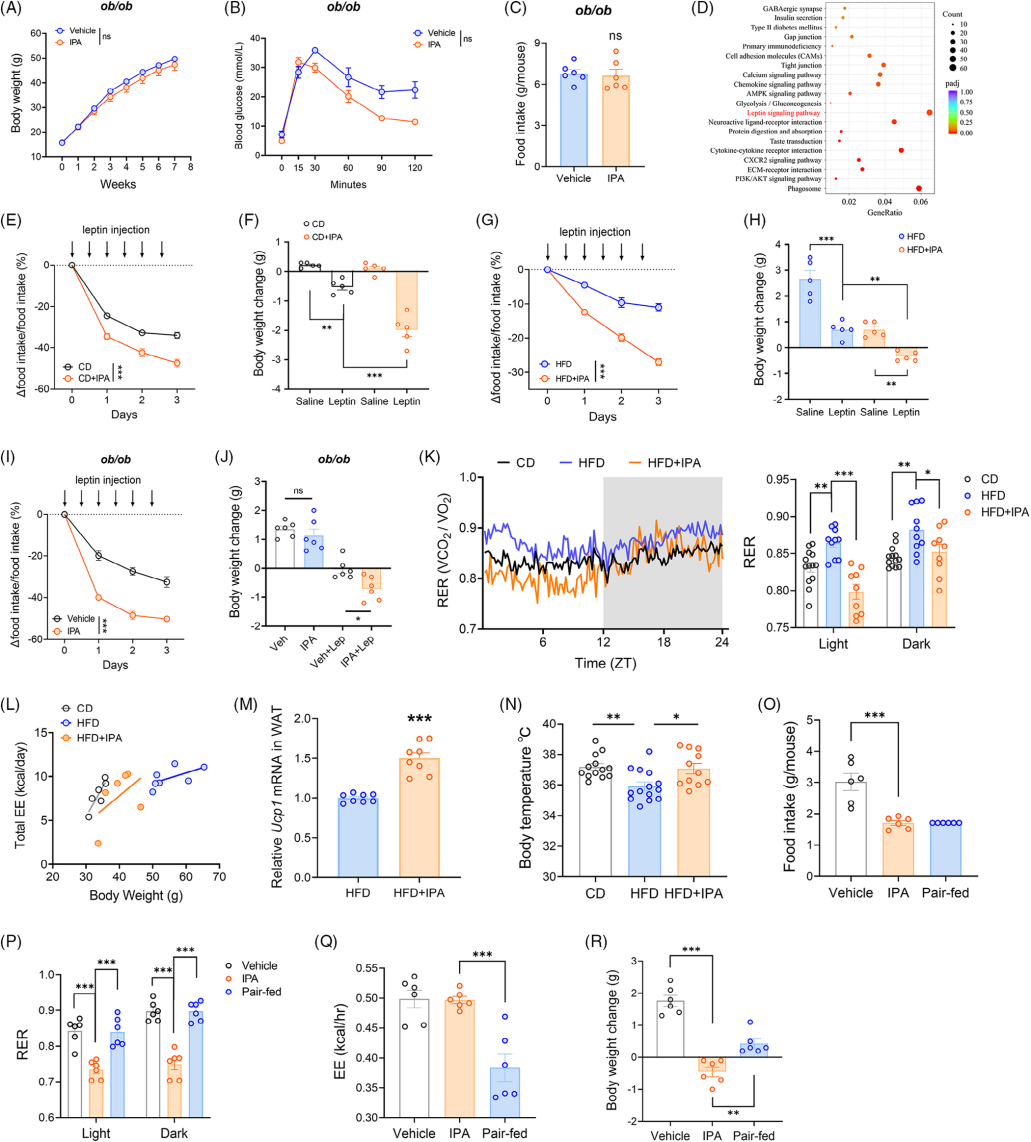
IPA is a leptin sensitiser. Body weight (A) and glucose tolerance (B) of ob/ob mice during the treatment. (*n* = 6 per group). (C) Average daily food intake of ob/ob mice at the end of the treatment period. (D) Hypothalamic differential gene pathway enrichment analysis of HFD mice treated with vehicle or IPA. (E, F) CD mice were pretreated with vehicle or IPA (20 mg/kg) for 1 week, followed by saline or leptin (1 mg/kg) injections for 3 days (*n* = 5 per group). Arrows indicate leptin injection. (E) Changes in daily food intake after vehicle or leptin injections. (F) Body weight changes following 3 days of vehicle or leptin injections. (G, H) HFD mice and HFD+IPA mice (treated with IPA for 16 weeks) were injected with saline or leptin (1 mg/kg) for 3 days (*n* = 5 per group). Arrows indicate leptin injection. (G) Changes in daily food intake following vehicle or leptin injections. (H) Body weight changes after 3 days of vehicle or leptin injections. (I, J) ob/ob mice were treated with either vehicle or IPA for 7 weeks, followed by saline or leptin (1 mg/kg) injections for 3 days (*n* = 6 per group). Arrows indicate leptin injection. (I) Changes in daily food intake following vehicle or leptin injections. (J) Body weight changes after 3 days of vehicle or leptin injections. (K) RER rhythm within 24 h in CD, HFD and HFD+IPA mice. The shaded area represents the active (dark) period. Bar charts represent the average RER values during the light and dark periods. CD group: *n* = 13; HFD group: *n* = 13; HFD+IPA group: *n* = 10. (L) EE over 24 h plotted against body weight. Each dot represents one mouse. (M) Measurement of *Ucp1* mRNA in WAT of vehicle and IPA‐treated HFD mice. (N) Body temperature of CD, HFD, and HFD+IPA mice (*n* = 11–14 per group). (O–R) Throughout the experimental period (7 days), DIO mice treated with either vehicle or IPA (20 mg/kg) were allowed ad libitum access to food, while the pair‐fed group had their food intake matched to that of the IPA‐treated group (*n* = 6 per group, all groups were fed a high‐fat diet). (O) Average daily food intake, (P) RER values, (Q) EE, and (R) body weight changes after the 7‐day experiment. Data were presented as mean ± SEM. Statistical significance was determined by Student's *t*‐test for C and M, by two‐way ANOVA for A, B, E, G and I, and by one‐way ANOVA for F, H, J, K, N, O and Q.

Next, we sought to determine whether IPA could treat genetically induced obesity in ob/ob mice. After 7 weeks of oral IPA administration (20 mg/kg, *i.g. qd*), no notable difference in weight gain was detected between the vehicle and IPA groups (Figure 7A). Additionally, there were no notable improvements in glucose tolerance or lipid levels (Figures 7B and S6D‐G), nor a significant alleviation of adipocyte degeneration (Figure S6H). We also observed that IPA treatment did not suppress the appetite of ob/ob mice (Figure 7C). Therefore, we hypothesise that the antiobesity effects of IPA depend on an intact leptin signalling pathway.

Leptin primarily targets the hypothalamic appetite regulation centre. We performed transcriptome sequencing on hypothalamic tissues from HFD mice treated with either IPA or vehicle. RNA‐Seq results revealed that differentially expressed genes following IPA treatment were significantly enriched in the leptin signalling pathway (Figure 7D). Leptin binding to its receptor (LepRb) activates downstream POMC and NPY/AGRP neurons, which suppress appetite and promote energy expenditure. We measured the mRNA levels of *POMC*, *NPY*, and *AGRP* in the hypothalamus and observed a significant increase in the expression of *POMC* and *NPY* significantly increased following IPA treatment, whereas *AGRP* mRNA levels remained unchanged (Figure S7A). Considering that *PTP1B* and *SOCS3* are negative feedback regulators of leptin signalling, we investigated the mRNA levels of *PTP1B* and *SOCS3* in the hypothalamus. Our results revealed that following IPA treatment, the expression level of *SOCS3* was significantly reduced, whereas the mRNA level of *PTP1B* remained unchanged (Figure S7B). These findings further support the potential role of IPA as a leptin sensitiser.

### 3.9 IPA is a leptin sensitiser

To investigate whether IPA functions as a bona fide leptin sensitiser, we compared the effects of exogenous leptin administration in IPA‐pretreated mice versus control mice. Initially, CD mice were pretreated with either a vehicle or IPA for 1 week, followed by leptin injection in each group. Leptin administration in CD mice significantly reduced both food intake and body weight, with IPA pretreatment markedly amplifying these effects (Figure 7E and F). It is important to note that IPA alone (IPA + Saline) did not result in significant body weight changes in CD mice (Figure 7F).

To assess if IPA could similarly enhance the effects of exogenous leptin in DIO mice, we examined the response of HFD‐fed mice to leptin with or without IPA treatment. HFD feeding induces leptin resistance in mice, hence leptin injection only slightly suppressed food intake and weight gain in these mice. However, IPA‐treated mice exhibited significant reductions in both food intake and body weight following leptin administration (Figure 7G and H). This finding indicates that IPA treatment enhances leptin sensitivity in HFD‐fed mice.

To elucidate IPA's role as a leptin sensitiser, we evaluated the impact of IPA pretreatment on the response to exogenous leptin in ob/ob mice. Administering leptin alone led to a significant reduction in both food intake and body weight in ob/ob mice. When combined with IPA, the anorexic and weight‐loss effects were even more pronounced than with leptin alone (Figure 7I and J). These results suggest that supplementation with exogenous leptin can restore IPA's therapeutic efficacy in ob/ob mice.

### 3.10 IPA improves energy metabolism balance in mice

It is well‐established that leptin enhances metabolic rate through thermogenesis.^26^, ^27^ Given that IPA can potentiate leptin's effects, we hypothesised that IPA would increase energy expenditure, leading to fat utilisation as a primary energy source. To test this, we measured metabolic parameters, including energy expenditure (EE) and respiratory exchange ratio (RER), in CD, HFD, and IPA‐treated mice. Notably, RER values significantly decreased in IPA‐treated mice during both light and dark cycles (Figure 7K). When analysing energy expenditure with body weight as a covariate, the IPA‐treated group exhibited a steeper slope of total energy expenditure relative to body weight compared to the HFD group (Figure 7L).

Further analysis revealed that IPA treatment elevated the expression of *Ucp1* mRNA in WAT, indicating enhanced thermogenic capacity in these mice (Figure 7M). Thermogenic rhythms are crucial for maintaining body temperature, and we observed higher body temperatures in the IPA‐treated group (Figure 7N). However, in ob/ob mice, RER values, EE, and body temperature were not directly affected by IPA treatment (Figure S8A‐C).

Subsequently, we analysed the metabolic rhythms among DIO mice treated with vehicle, IPA, and those in the pair‐fed group. During the study, both the vehicle and IPA groups had unrestricted access to food, while the pair‐fed group received a controlled amount of food to match the intake of the IPA group (Figure 7O). Throughout both light and dark phases, the IPA‐treated mice exhibited significantly lower RER values compared to the vehicle and pair‐fed groups (Figure 7P). The energy expenditure (EE) in the IPA‐treated group was substantially higher than in the pair‐fed group (Figure 7Q). Moreover, the weight reduction in the IPA‐treated group was significantly greater than that in the vehicle and pair‐fed groups (Figure 7R). These results underscore the role of IPA in regulating energy balance.

### 3.11 STAT3 is a direct binding protein for IPA

**FIGURE 8:**
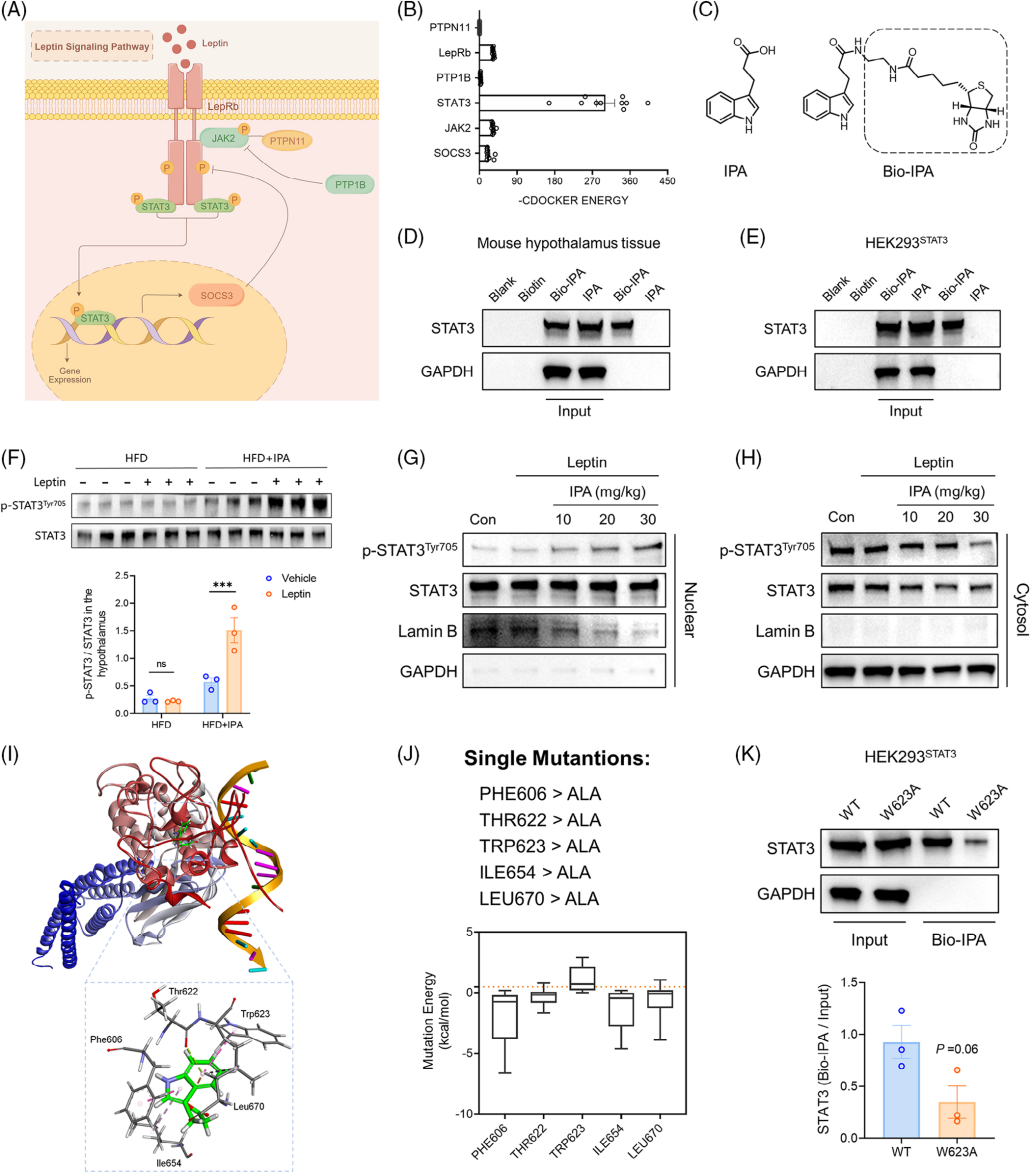
IPA targets and binds to STAT3 protein, enhancing leptin signalling. (A) Schematic representation of the leptin signalling pathway. (B) The CDOCKER scores for the molecular docking of IPA with the six key proteins involved in leptin signal transduction. (C) Chemical structures of IPA and biotin‐labelled IPA (Bio‐IPA). (D, E) Incubate Bio‐IPA with streptavidin magnetic beads, using biotin and IPA as controls. Add protein lysates extracted from mouse hypothalamus tissue (D) and HEK293 cells transfected with hSTAT3 plasmid (E). Perform Western blot analysis thereafter. Total lysates are used as input. (F) Western blot showing the impact of acute leptin stimulation on phosphorylation of STAT3 (Y705) in the hypothalamus. The data in the figure represent the quantified ratio of p‐STAT3/STAT3. (G, H) IPA enhances leptin‐mediated STAT3 nuclear translocation in a dose‐dependent manner. Following treatment of HFD mice with varying doses of IPA (10, 20, and 30 mg/kg) for 6 weeks, exogenous leptin was administered, and nuclear (G) and cytoplasmic (H) proteins were immediately extracted from the hypothalamic tissues. Western blot analysis was conducted to assess p‐STAT3 and STAT3 levels. Lamin B and GAPDH served as markers for nuclear and cytoplasmic proteins, respectively. (I) A schematic representation of the binding between IPA and the SH2 domain of the STAT3 protein. The enlarged view highlights the amino acid residues involved in the interaction. (J) Virtual alanine mutagenesis was performed on five amino acid residues involved in the interaction, replacing each residue with alanine (Ala) and calculating the mutation energy. (K) HEK293 cells were transfected with plasmids encoding either wild‐type STAT3 or mutant STAT3 (W623A). Cell lysates were then extracted for pull‐down assays. Data were presented as mean ± SEM. Statistical significance was determined by Student's *t*‐test for F and K.

Upon activation of its membrane‐bound receptor, leptin triggers a cascade of intracellular signalling events. This process primarily involves the phosphorylation of JAK2, which subsequently initiates downstream signalling pathways. STAT3 is recruited to LepRb, where it becomes phosphorylated, translocates to the nucleus, and activates the transcription of target genes, including SOCS3. Both SOCS3 and PTP1B act as negative feedback regulators, ensuring that LepRb activation does not exceed physiological requirements (Figure 8A).

Given that IPA can enhance leptin's effects, we hypothesised that it likely interferes with the leptin signalling pathway. To identify the specific step affected by IPA, we conducted molecular docking studies using the crystal structures of six key proteins involved in leptin signal transduction. The CDOCKER algorithm, based on the CHARMm force field, was employed to generate high‐precision docking results.^28^, ^29^ Surprisingly, the CDOCKER score for the STAT3 protein was the highest, indicating a significantly strong affinity for IPA, far exceeding that of the other proteins (Figure 8B).

To verify the direct interaction between IPA and STAT3 proteins, we employed a biotinylated small molecule pull‐down assay. Biotin‐labelled IPA (Bio‐IPA; Figure 8C) was added to streptavidin magnetic beads, followed by the addition of mouse hypothalamic tissue lysate. The results indicated that Bio‐IPA could bind to the STAT3 protein in the mouse hypothalamic tissue lysate (Figure 8D). Furthermore, transfected human STAT3 protein (hSTAT3) in HEK293 cells was also pulled down by Bio‐IPA (Figure 8E). These results provide additional evidence that IPA can directly bind to STAT3.

### 3.12 IPA enhances leptin‐mediated STAT3 activation

The interaction between IPA and STAT3 prompted us to investigate whether IPA could influence STAT3 activation, thereby potentiating leptin's effects. We used tyrosine 705 phosphorylation of STAT3 as a marker of STAT3 activation. As expected, HFD induced leptin resistance in mice, and exogenous leptin administration failed to increase hypothalamic phospho‐STAT3 (Y705) levels. In contrast, IPA‐treated mice exhibited enhanced STAT3 phosphorylation, which was further elevated following leptin injection (Figure 8F).

Given that the nuclear translocation of STAT3 is a critical event in STAT3‐mediated gene regulation, we isolated nuclear and cytoplasmic proteins from the hypothalamic tissues of HFD mice treated with varying doses of IPA (10, 20, and 30 mg/kg) for 6 weeks. Our findings revealed that IPA augmented leptin‐mediated STAT3 nuclear translocation in a dose‐dependent manner (Figure 8G and H). Thus, IPA regulates STAT3 activity through the promotion of both phosphorylation and nuclear translocation.

### 3.13 Binding mode of IPA to STAT3

We further analysed the interaction sites between IPA and the STAT3 protein (PDB: 1BG1). The highest‐scoring conformation from CDOCKER simulations revealed that IPA binds to the Src‐homology‐2 (SH2) domain of STAT3 (Figure 8I). The amino acid residues on STAT3 involved in the interaction with IPA include Phe606, Thr622, Trp623, Ile654, and Leu670. We performed virtual alanine scanning mutagenesis on these five residues, replacing each with alanine (Ala) and calculating the mutation energy to identify key residues within the active site (Figure 8J). The results indicated that only the average mutation energy of Trp623 mutation (Trp623Ala, W623A) exceed .5, suggesting that this mutation has a destabilising effect on the protein‐ligand complex. This implies that the W623A mutation reduces the affinity between STAT3 and IPA, weakening their interaction.

Subsequently, we constructed a mutant plasmid encoding STAT3^W623A^ based on pCMV3‐Flag‐hSTAT3 and transfected them into HEK293 cells. We then performed pull‐down assays to determine whether Bio‐IPA binds to STAT3 in the transfected cells. The results showed that Bio‐IPA effectively bound to the wild‐type STAT3 protein in the cell lysates, whereas the binding to the STAT3^W623A^ mutant was significantly reduced (Figure 8K). These findings indicate that Trp623 in the SH2 domain is a critical residue for the interaction between IPA and STAT3.

## 4 DISCUSSION

In recent years, there have been considerable advancements in obesity treatment, yet therapies targeting its fundamental processes remain lacking. Recently, GLP‐1 receptor agonists have been approval for application in type 2 diabetes and obesity.^30^, ^31^ However, these drugs are typically injectable and expensive, limiting their accessibility to some patients. Here, we employed a strategy that seeks potential treatments from body itself, leveraging the inherent safety profile of endogenous small molecules. This approach posits that in diet‐induced obesity, the body's metabolic system undergoes changes, and those metabolites that significantly decrease in concentration under obese conditions may hold potential as antiobesity agents. Ultimately, we identified a highly effective antiobesity molecule derived from gut microbiota, IPA.

IPA is a derivative of indole, the production of which in humans and mice is entirely dependent on gut microbiota.^19^ Consequently, the concentration of such substances in the body is influenced by the composition of the gut microbial community. In addition to IPA, we also observed a decrease in the levels of indoxyl sulfate and 5‐methoxyindoleacetate in DIO mice (Figure 1C). Tryptophan serves as the common metabolic precursor for these three compounds. Indoxyl sulfate, considered a harmful metabolite produced by gut microbiota, is a uremic toxin that is associated with chronic kidney disease.^32^, ^33^ Unlike IPA, indoxyl sulphate does not exhibit beneficial metabolic effects, as demonstrated by our findings (Figure 5B). 5‐Methoxyindoleacetate is a product of serotonin metabolism, and its reduction re‐emphasises the impact of a high‐fat diet on gut microbiota function and tryptophan metabolism.^34^ The metabolic accumulation of IPA and 5‐methoxyindoleacetate in vivo is dependent on *C. sporogenes*, whereas indoxyl sulfate is reliant on *Bacteroides thetaiotaomicron*.^20^ We observed that HFD leads to a reduction in both the quantity and diversity of gut microbiota, consistent with previous research findings.^35^, ^36^ We further elucidated the underlying mechanism, revealing that HFD abnormally activates Paneth cells through the IFNγ‐Irgm1 axis, causing an excessive release of antimicrobial peptides that inhibit the growth of gut microbiota, resulting in an overall decrease in indole derivatives.

The regulation of energy balance is managed by the central nervous system, which integrates satiety cues along with obesity‐related signals like leptin and insulin.^37^ However, chronic intake of calories exceeding daily energy expenditure leads to increased fat accumulation and elevated circulating leptin levels, resulting in reduced neuronal responsiveness to leptin.^9^, ^38^ This hypothalamic leptin resistance limits the direct therapeutic application of leptin for obesity. STAT3 is essential in leptin signalling and plays a key role in regulating energy balance.^8^ We found that IPA, after being secreted from the gut into the bloodstream, reaches the central nervous system and directly binds to hypothalamic STAT3 protein. This binding promotes STAT3 phosphorylation and nuclear translocation, thereby enhancing leptin signal transduction. Consequently, we propose that IPA functions as a leptin sensitiser. The physiological and biochemical reactions observed in mice following exogenous leptin administration provide evidence supporting this hypothesis. When leptin was injected, despite leptin resistance in DIO mice, IPA enhanced the responsiveness to leptin, as evidenced by decreased appetite and body weight and increased phosphorylation levels of STAT3 at Tyr705. Critically, IPA was ineffective in ob/ob mice, indicating that its action depends on the integrity of the leptin signalling pathway; however, the leptin‐sensitising effect of IPA was restored when exogenous leptin was administered to ob/ob mice. Additionally, HFD mice treated with IPA showed significantly higher EE compared to pair‐fed controls, further suggesting increased leptin sensitivity. Taken together, these findings provide strong evidence that IPA counteracts obesity by enhancing leptin sensitivity.

IPA is considered to have antioxidant properties and potential benefits in reducing oxidative stress,^39^, ^40^ but it is also important to concern any potential adverse effects. Based on the current 16‐week data from mice, no adverse effects have been observed. IPA seems to have no notable effects on the mental state or liver and kidney function in mice, suggesting a relatively high safety profile. However, whether long‐term supplementation of IPA might result in adverse effects requires further investigation in human studies.

**FIGURE 9:**
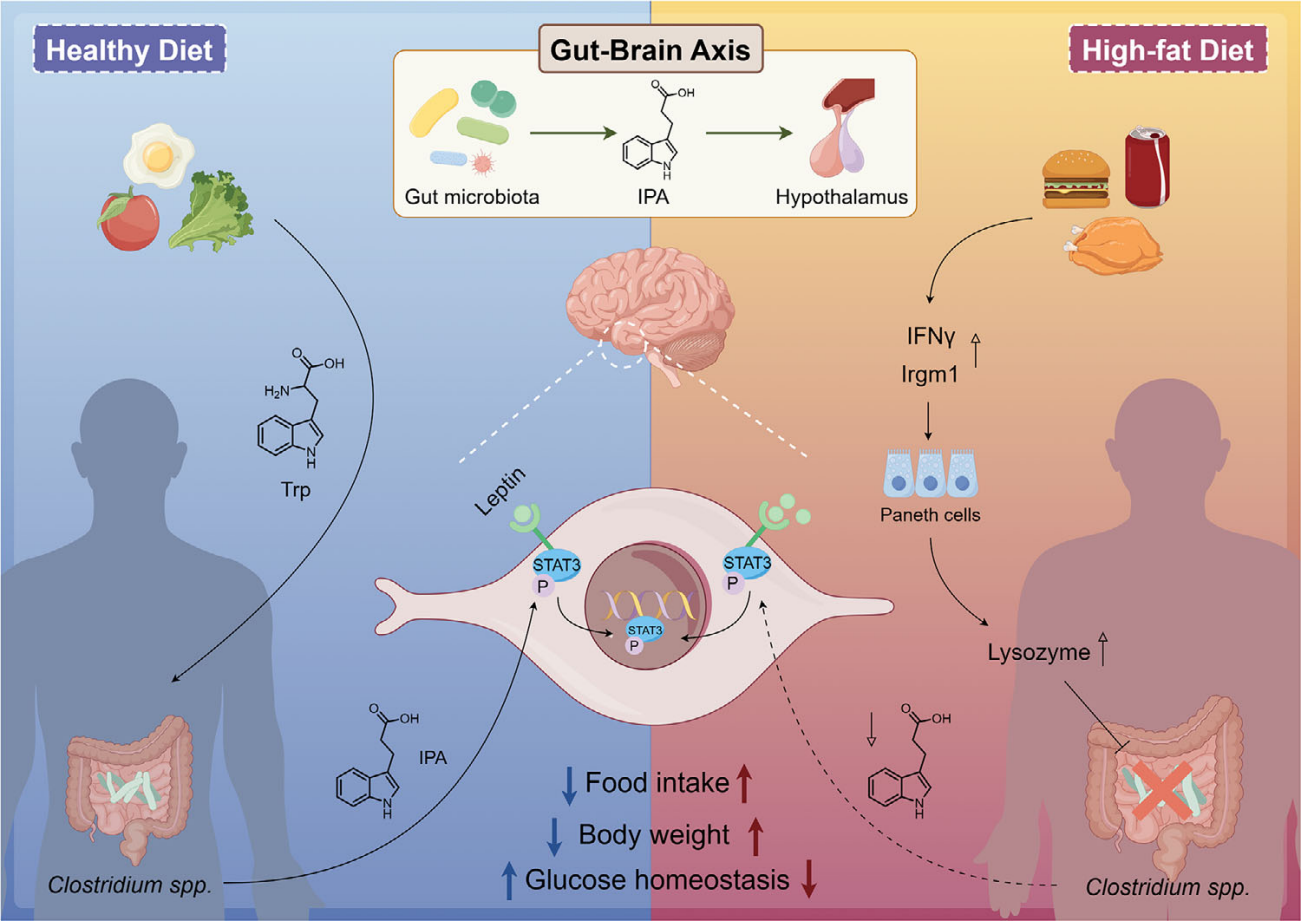
Schematic illustration shows the metabolic cycle of IPA in the body and its role as a leptin sensitiser.

Our research offers a novel perspective, demonstrating that the gut microbiota‐derived metabolite, IPA, can enhance the action of leptin, a hormone secreted by adipocytes (Figure 9). This finding highlights the collaborative relationship between endogenous metabolic regulators, even though the endogenous factors are not conventionally produced solely by human cells. Gut microbiota is currently recognised as a crucial ‘invisible organ’ in the human body. Viewing the gut microbiome as an essential component of the human body contributes to elucidating its pivotal role in maintaining overall health and managing disease. Investigating the interactions between the gut microbiome and the human host not only enhances our understanding of this vital system but also uncovers new opportunities for therapeutic interventions and health optimisation.

## AUTHOR CONTRIBUTIONS

**Xin Ma and Zhiwei Wang**: conceived the project. **Zhiwei Wang and Shaying Yang**: performed experiments and interpreted all the results. **Zhiwei Wang; Tingting Zhou; and Liangju Liu**: contributed to the recruitment of participants. **Aiqin Mao and Hao Kan**: provided experimental advice. **Lei Feng and Fan Yu**: contributed to the animal housekeeping. **Zhiwei Wang**: wrote the manuscript. All authors discussed the results and approved the manuscript.

## CONFLICT OF INTEREST STATEMENT

The authors declare no conflicts of interest.

## ETHICS STATEMENT

All animal experiments were conducted in accordance with the approved protocol by the Jiangnan University Animal Care Committee, with the specific protocol number JN. No 20221215t0160730[560]. The recruitment of participants and sample collection were approved by the Ethics Committee of Jiangnan University (Approval Number: JNU20200109IRB04). The participants included in this study for analysis were the obese subgroup cohort under this approved ethical protocol. All participants provided informed consent.

## Supporting information



Supporting information

## Data Availability

The data that support the findings of this study are available from the authors upon reasonable request.
