# Supplementary material for: The gut microbiota‐derived metabolite indole‐3‐propionic acid enhances leptin sensitivity by targeting STAT3 against diet‐induced obesity
Source: Clin Transl Med. 2024 Nov 28;14(12):e70053. doi: 10.1002/ctm2.70053 (PMC11602751; doi:10.1002/ctm2.70053)
Supplement: Supplementary file 1 — Supporting information [file CTM2-14-e70053-s001.docx]

**Supplemental Materials** **and Methods**

**The gut microbiota-derived metabolite indole-3-propionic acid enhances leptin sensitivity by targeting STAT3 against diet-induced obesity**

Zhiwei Wang^1,#^, Shaying Yang^1,^**^#^**, Tingting Zhou^1^, Liangju Liu^1^, Aiqin Mao^1^, Hao Kan^1^, Lei Feng^1^, Fan Yu^1^, Xin Ma^1,2,^*

^1^ Department of Pharmacology, Wuxi School of Medicine, Jiangnan University, Wuxi, China

^2^ Medical Basic Research Innovation Center for Gut Microbiota and Chronic Diseases, Wuxi School of Medicine, Jiangnan University, Wuxi, China

**^#^** These authors contributed equally to this study

* Correspondence

Tel: 86-510-85914599 (Xin Ma)

Email address: [maxin@jiangnan.edu.cn](mailto:maxin@jiangnan.edu.cn) (Xin Ma)

**Supplemental Figures and Figure Legends**

**Figure S1**

**
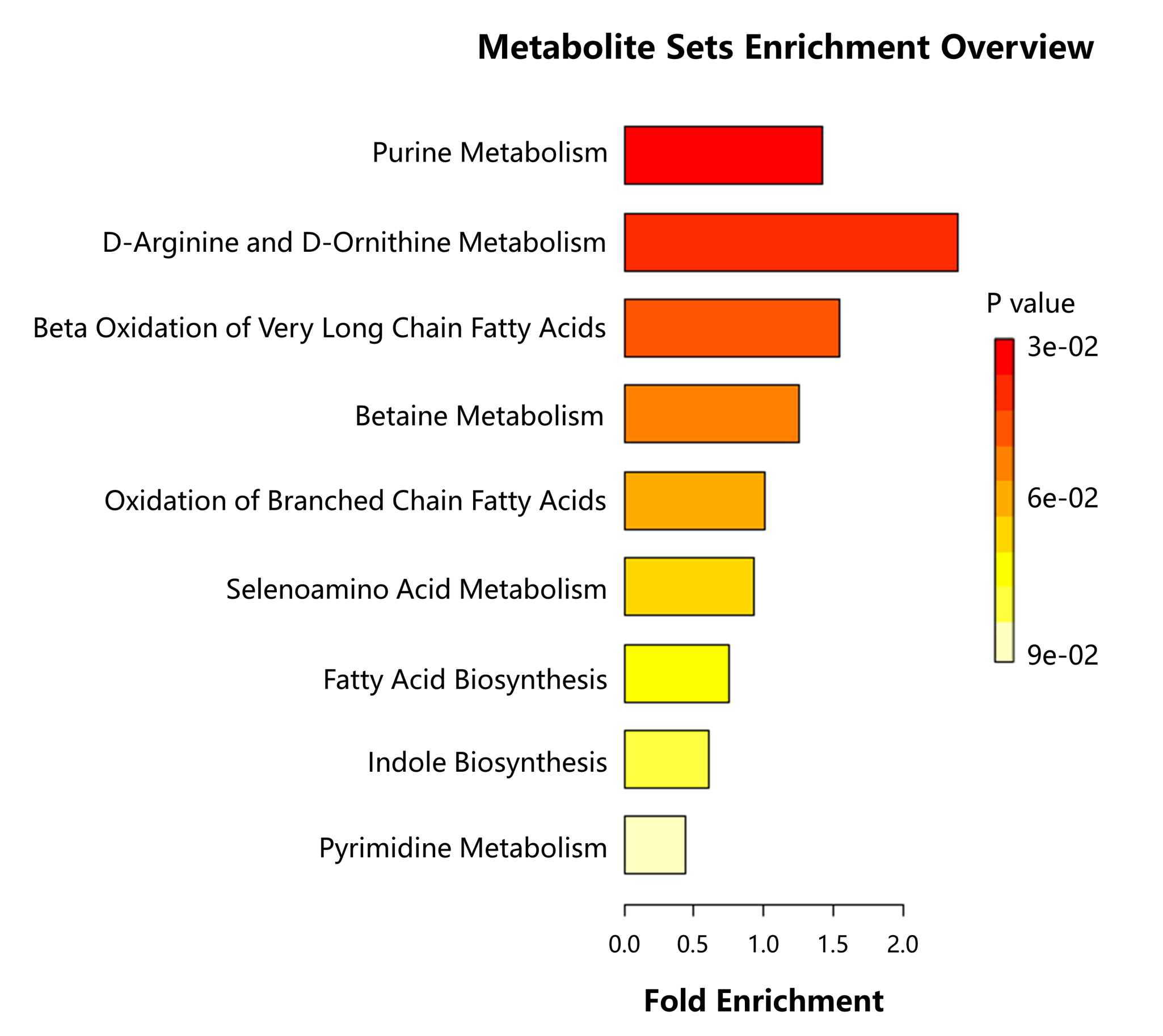
**

**Figure S1. Summary Plot for Over Representation Analysis (ORA).** Analysis of distinct enrichment pathways for serum metabolites in mice fed HFD.

**Figure S2**


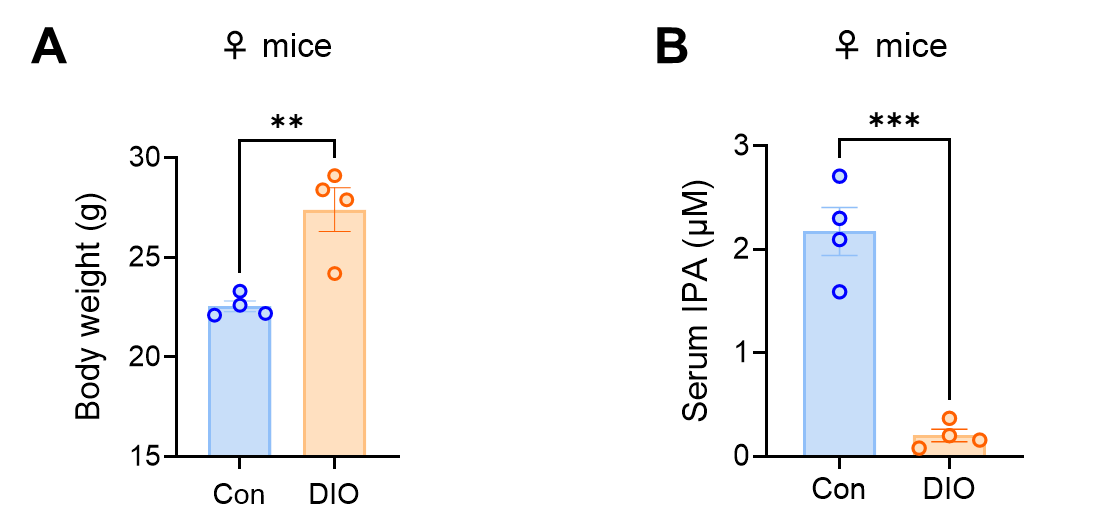


**Figure S2.** Comparison of body weight (**A**) and serum IPA levels (**B**) between female control mice and DIO mice (n = 4 mice per group). Data were presented as mean ± SEM. Statistical significance was determined by Student’s t-test for **A** and **B**.

**Figure S3**

**
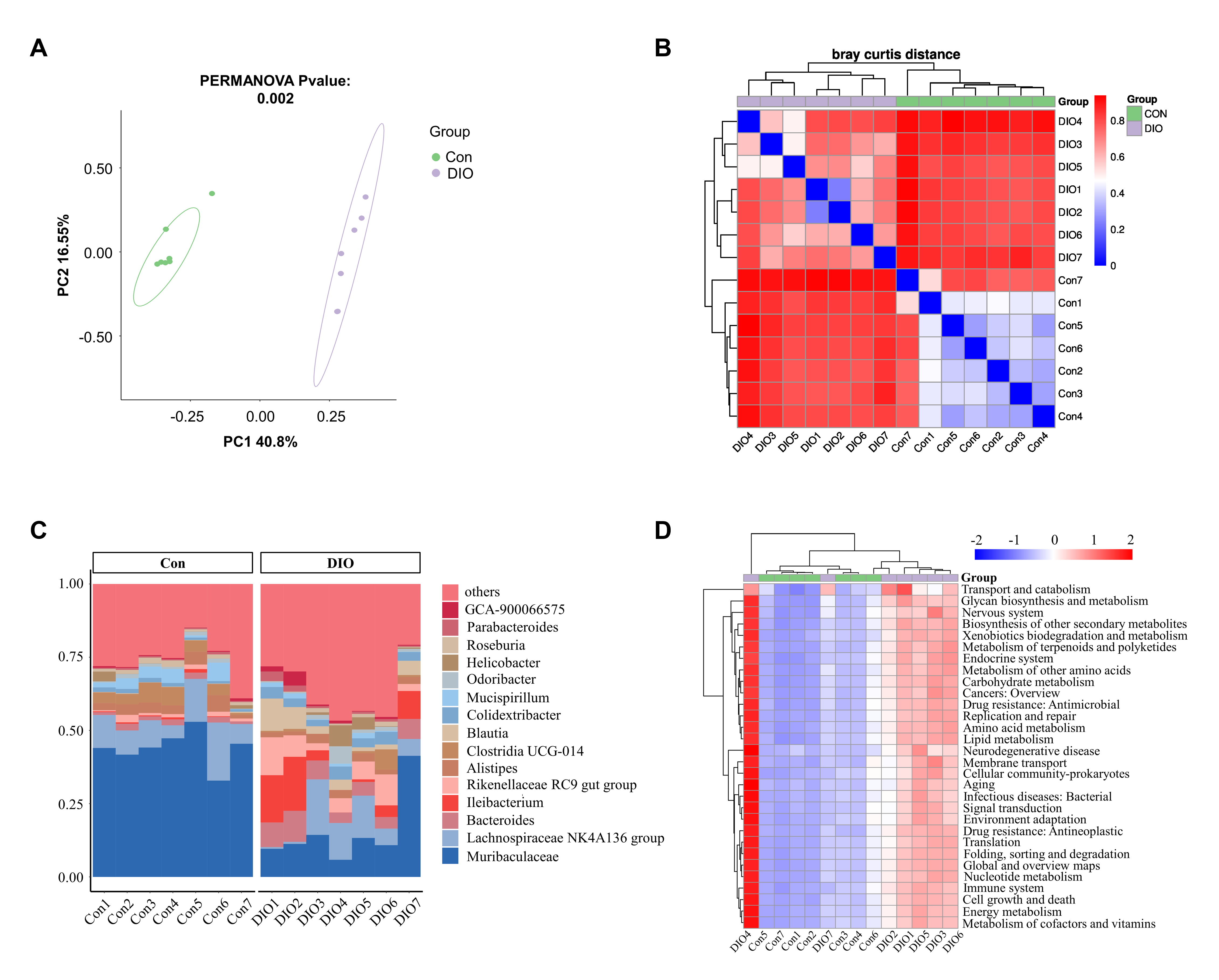
**

**Figure S3.** **Microbiota Analysis.** (**A**) Principal Coordinate Analysis (PCoA) illustrates the differences in the gut microbiome composition of healthy control mice (n=7) and DIO mice (n=7) based on fecal samples. (**B**) Bray-Curtis similarity heatmap of gut microbiota between the two groups. Taxonomic composition (**C**) and metabolic functions (**D**) of fecal microbiota in healthy control mice and DIO mice.

**Figure S4**


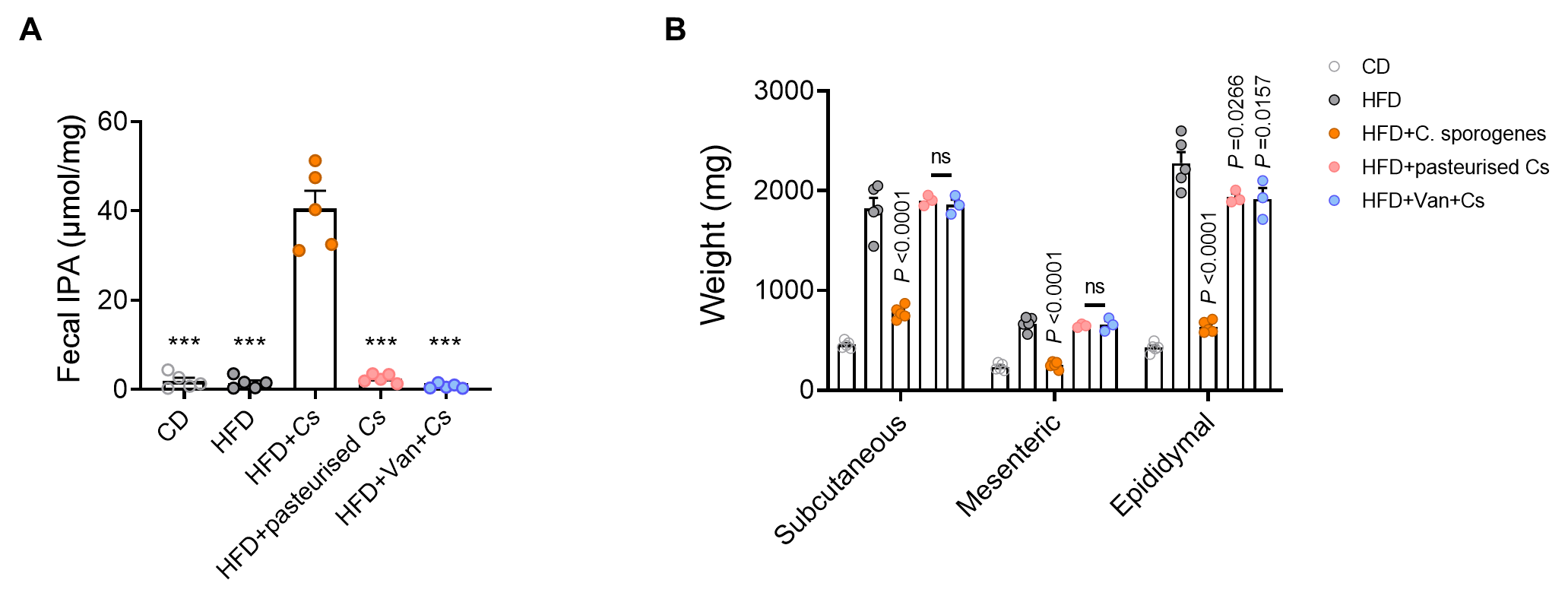


**Figure S4.** (**A**) Levels of IPA in the feces of mice from each group. (**B**) Mass of different fat pads, namely subcutaneous, mesenteric, and epididymal fat, in mice from each group. Data were presented as mean ± SEM. Statistical significance was determined by one-way ANOVA.

**Figure S5**

**
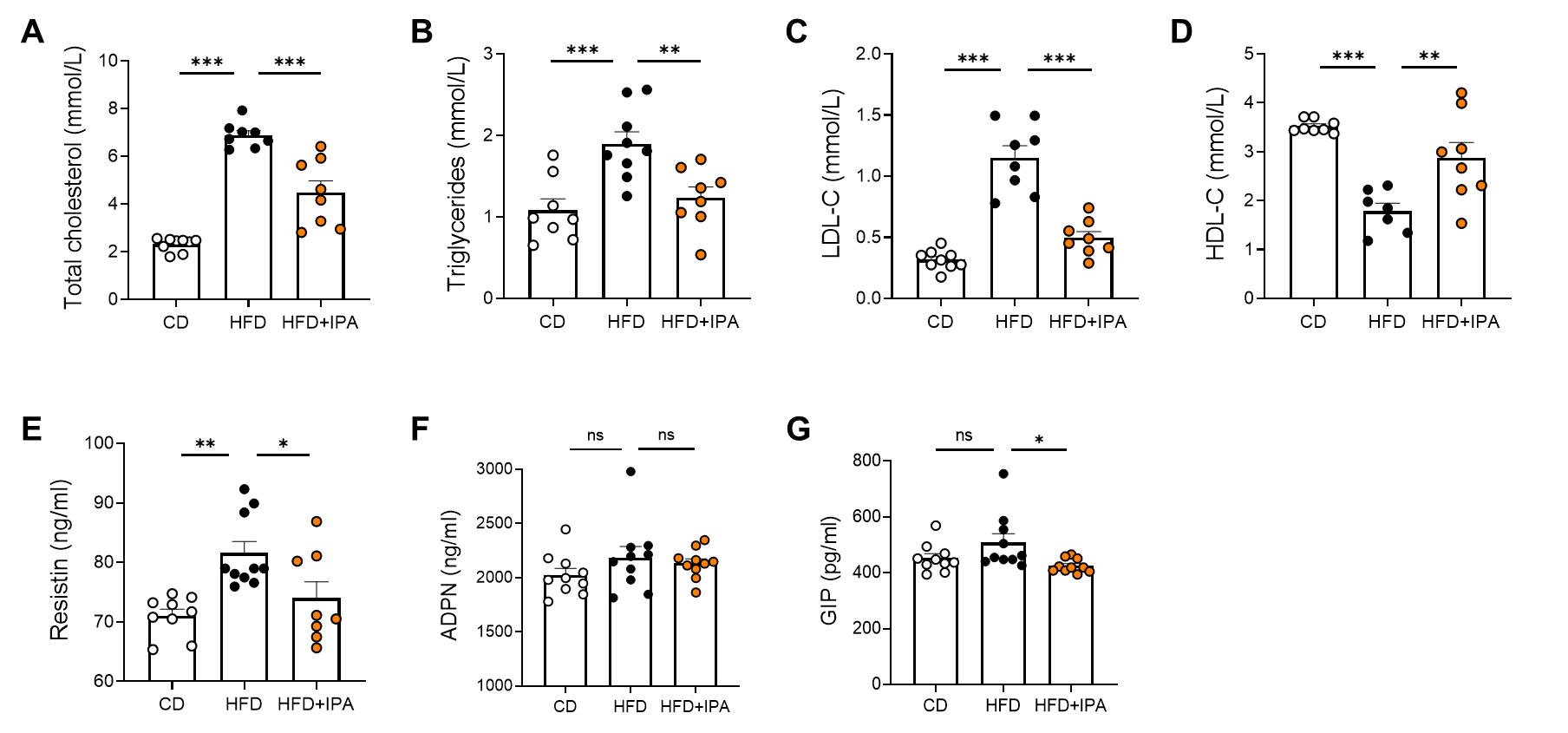
**

**Figure S5.** **IPA improves lipid metabolism in HFD mice.** Serum levels of total cholesterol (**A**), triglycerides (**B**), LDL-C (**C**), HDL-C (**D**), resistin (**E**), ADPN (**F**), and GIP (**G**) following a 6-hour fasting period. Data were presented as mean ± SEM. Statistical significance was determined by one-way ANOVA for all.

**Figure S6**


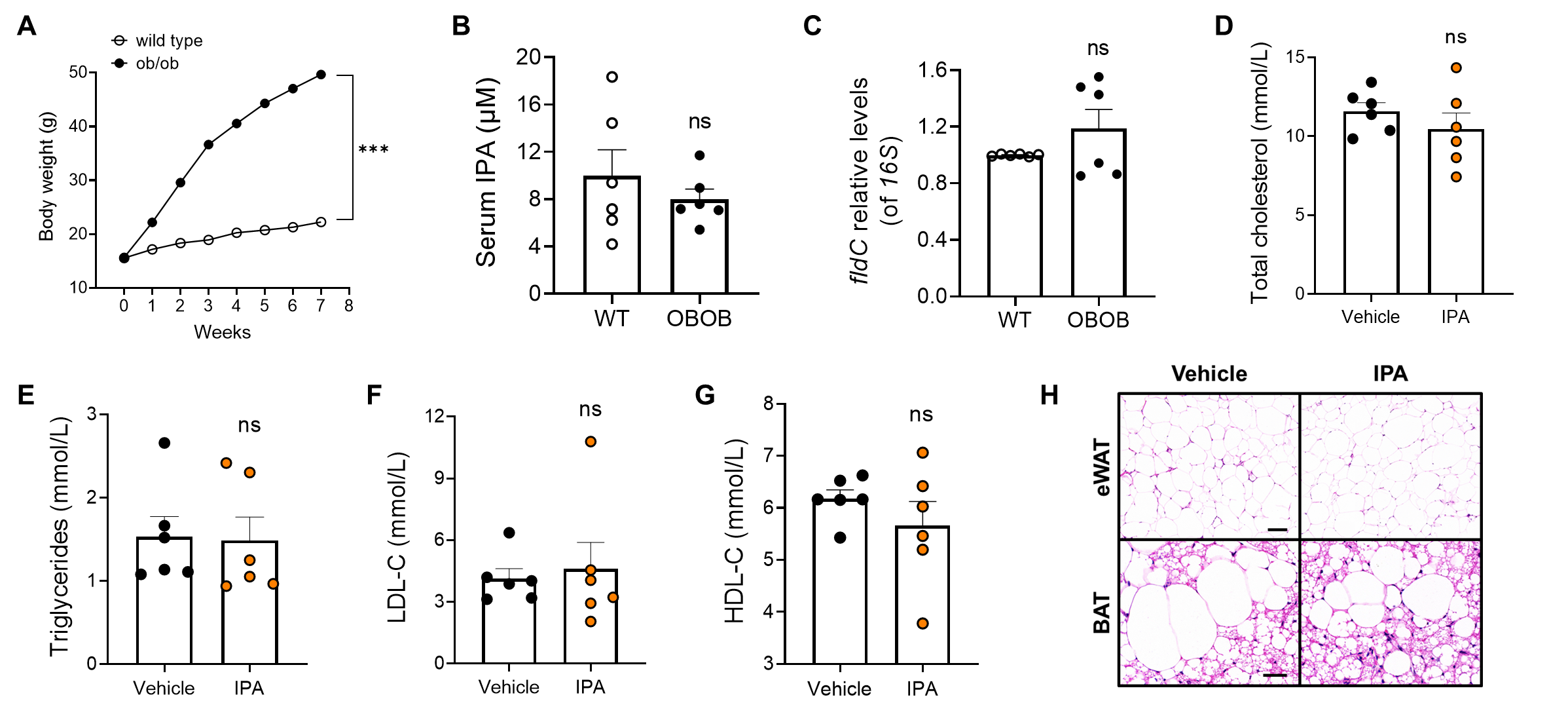


**Figure S6. The anti-obesity effects of IPA depend on intact leptin signaling.** Comparison of the body weight (**A**), serum IPA levels (**B**), and fecal fldC levels (**C**) between ob/ob mice and age-matched wild-type mice (n = 6 per group). After 7 weeks of oral gavage with IPA (20 mg/kg) or vehicle, the levels of total cholesterol (**D**), triglycerides (**E**), LDL-C (**F**) and HDL-C (**G**) in the serum of ob/ob mice were assessed (n = 6 per group). (**H**) Representative images of H&E staining in eWAT and BAT. Scale bar: 100 µm. Data were presented as mean ± SEM. Statistical significance was determined by Student’s *t*-test for **B**, **C**, **D, E, F** and **G**, and by two-way ANOVA for **A**.

**Figure S7**


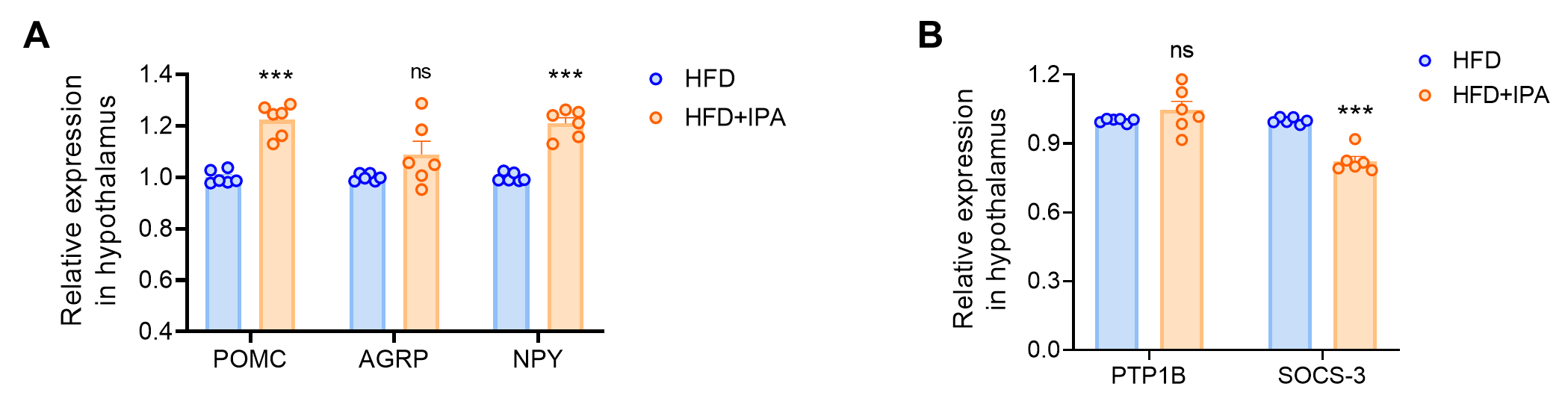


**Figure S7.** After IPA treatment, the relative expression levels of *POMC*, *AGRP*, and *NPY* (**A**), *PTP1B* and *SOCS-3* (**B**) in the hypothalamus of HFD mice (n = 6 per group). Data were presented as mean ± SEM. Statistical significance was determined by Student’s *t*-test.

**Figure S8**


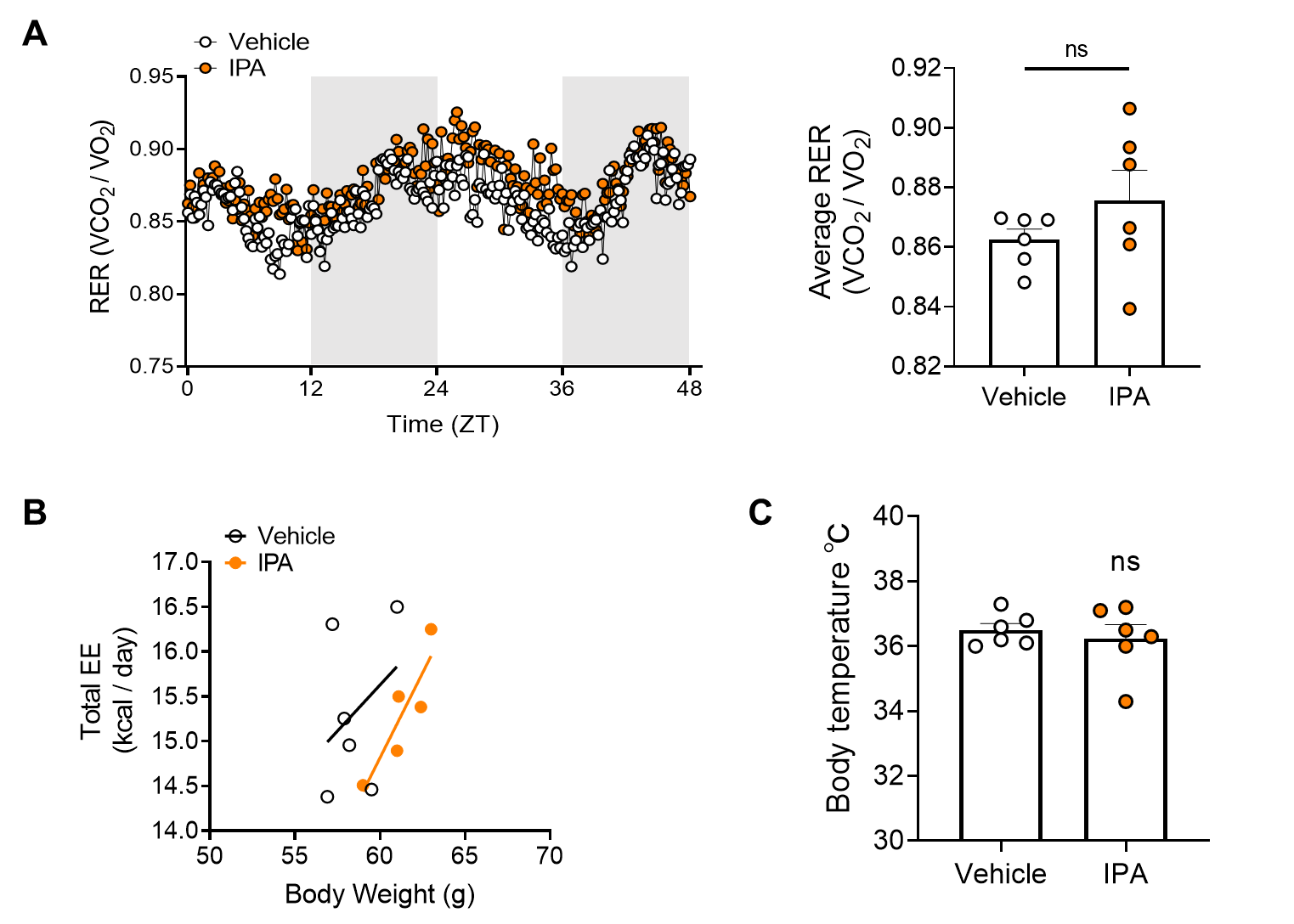


**Figure S8. IPA cannot enhance the metabolism of ob/ob mice.** (**A**) RER rhythm and average VCO_2_/VO_2_ levels within 24 hours in ob/ob mice. The shaded area represents the active (dark) period. (**B**) Total energy expenditure (EE) over 24 hours plotted against body weight in ob/ob mice. Each dot represents one mouse. (**C**) Body temperature of ob/ob mice. Data were presented as mean ± SEM. Statistical significance was determined by Student’s *t*-test for **A** and **C**.

**Figure S9**

**
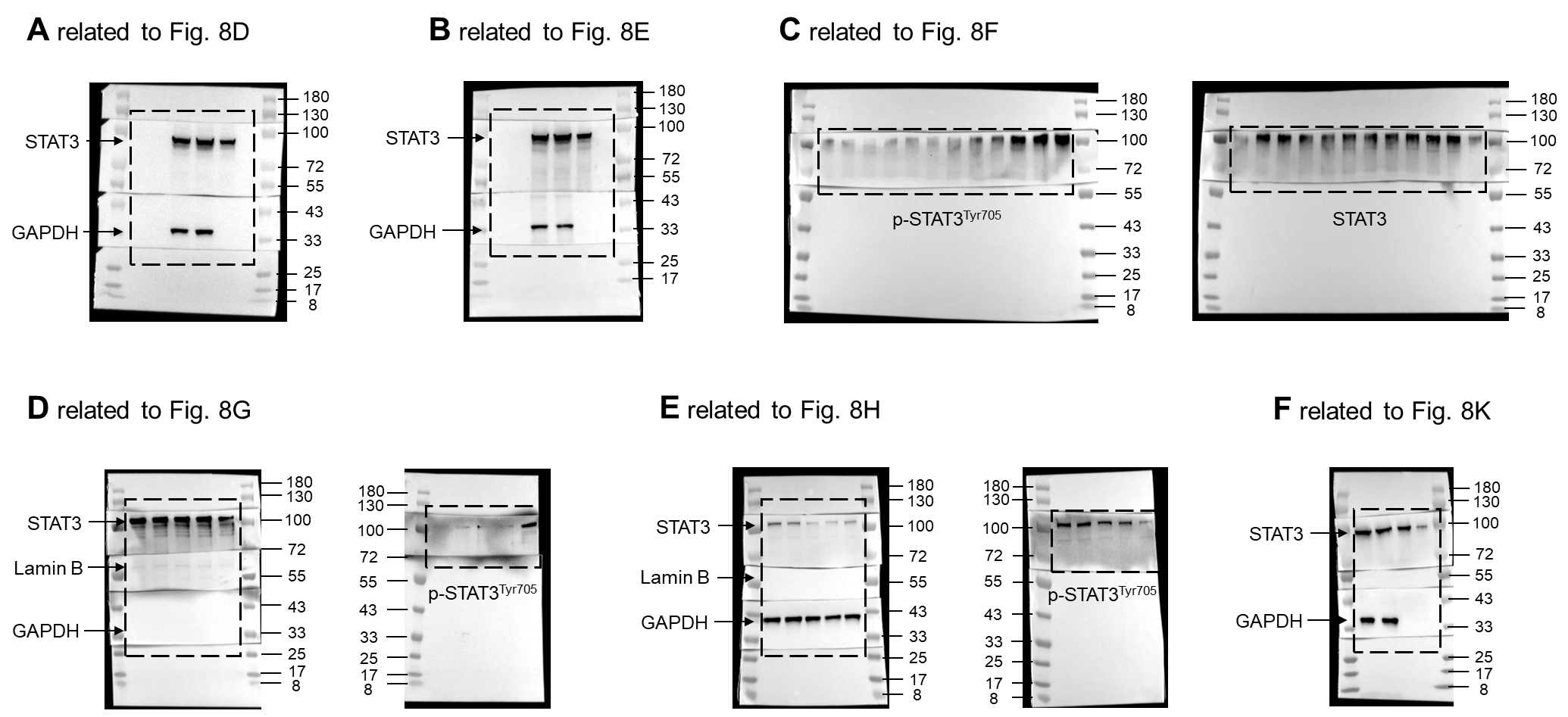
**

**Figure S9. Whole uncropped images of the original Western Blot.** (**A-F**) Whole uncropped images of the original WB related to representative images in the Fig. 8D, E, F, G, H and K.

**Supplemental Tables**

**Supplemental Table 1.** Baseline characteristics of the participants

| Characteristics | Lean (n = 21) | Obese (n = 21) | *P* Value |
| --- | --- | --- | --- |
| Age, y | 40.7 ± 7.9 | 43.3 ± 8.6 | 0.286 |
| Male, n (%) | 12 (57.1) | 14 (66.7) | 0.525 |
| Body mass index, kg/m^2^ | 21.1 ± 2.9 | 29.4 ± 1.7 | ＜0.001 |
| Systolic blood pressure, mmHg | 115.1 ± 9.2 | 120.6 ± 11.5 | 0.0977 |
| Total cholesterol, mmol/L | 3.33 ± 0.7 | 4.9 ± 0.9 | ＜0.001 |
| Triglycerides, mmol/L | 1.0 ± 0.4 | 2.6 ± 1.6 | ＜0.001 |
| Blood glucose, mmol/L | 7.3 ± 1.6 | 7.8 ± 2.2 | 0.384 |
| Current smoker, n (%) | 4 (19.0) | 6 (28.6) | 0.469 |
| Anti-obesity drugs, n (%) | 0 | 0 |  |
| Antibiotic, n (%) | 0 | 0 |  |

All participants were of Asian Han ethnicity. Data are presented as mean ± standard deviation or as numbers and percentages (%). Non-paired t-tests were used for the comparison of continuous variables with a normal distribution between the two groups. The χ² test were employed for comparing categorical variables.

**Supplemental Table 2.** Baseline characteristics of FMT donors

| Characteristics | Lean donors (n = 5) | Obese donors (n = 7) | *P* Value |
| --- | --- | --- | --- |
| Age, y | 41.0 ± 2.0 | 44.6 ± 6.1 | 0.239 |
| Male, n (%) | 3 (60.0) | 3 (42.9) | 0.558 |
| Body mass index, kg/m^2^ | 17.7 ± 1.3 | 31.3 ± 1.2 | ＜0.001 |
| Systolic blood pressure, mmHg | 108.0 ± 2.6 | 113.4 ± 8.9 | 0.2224 |
| Total cholesterol, mmol/L | 3.3 ± 0.3 | 5.2 ± 0.9 | 0.0016 |
| Triglycerides, mmol/L | 1.0 ± 0.5 | 3.5 ± 1.6 | 0.006 |
| Blood glucose, mmol/L | 6.7 ± 0.9 | 8.8 ± 2.8 | 0.142 |
| Current smoker, n (%) | 1 (20.0) | 2 (28.6) | 0.735 |
| Anti-obesity drugs, n (%) | 0 | 0 |  |
| Antibiotic, n (%) | 0 | 0 |  |

All participants were of Asian Han ethnicity. Data are presented as mean ± standard deviation or as numbers and percentages (%). Non-paired t-tests were used for the comparison of continuous variables with a normal distribution between the two groups. The χ² test were employed for comparing categorical variables.

**Supplemental Table 3.** Sequences of primers for real-time quantitative PCR

| Gene | Forward primer (5' to 3') | Reverse primer (5' to 3') |
| --- | --- | --- |
| TBP | GAAGCTGCGGTACAATTCCAG | CCCCTTGTACCCTTCACCAAT |
| Cd36 | ATGGGCTGTGATCGGAACTG | GTCTTCCCAATAAGCATGTCTCC |
| Fatp4 | GGCTCTCATCAACACCAACC | CCAGGATCCAGAGCAGAAGA |
| Lpl | TCTGTACGGCACAGTGG | CCTCTCGATGACGAAGC |
| Atgl | ACAGCTCCAACATCCAC | AGCCCTGTTTGCACATCTCT |
| Hsl | GCTAGCCAGGCTCATCTCCT | GTTCTTGAGGTAGGGCTCGT |
| Plin1 | GATGCCCTGAAGGGTGTTAC | CCTCTGCTGAAGGGTTATCG |
| SCD1 | TTCTTGCGATACACTCTGGTGC | CGGGATTGAATGTTCTTGTCGT |
| Tnfα | TCGAGTGACAAGCCTGTAGCC | TTGAGATCCATGCCGTTGG |
| IL-1β | TGTGAATGCCACCTTTTGACA | GGTCAAAGGTTTGGAAGCAG |
| IL-6 | TAGTCCTTCCTACCCCAATTTCC | TTGGTCCTTAGCCACTCCTTC |
| Mcp1 | GCAGTTAACGCCCCACTCA | TCCAGCCTACTCATTGGGATCA |
| F4/80 | TGACAACCAGACGGCTTGTG | GCAGGCGAGGAAAAGATAGTGT |
| Cd68 | CTTCCCACAGGCAGCACAG | AATGATGAGAGGCAGCAAGAGG |
| Cd163 | TCCACACGTCCAGAACAGTC | CCTTGGAAACAGAGACAGGC |
| Cd11c | ACGTCAGTACAAGGAGATGTTGGA | ATCCTATTGCAGAATGCTTCTTTACC |
| Cd11b | GTCAGAGTCTGCCTCCGTGT | CCTGCGTGTGTTGTTCTTTG |
| Lbp | GTCCTGGGAATCTGTCCTTG | CCGGTAACCTTGCTGTTGTT |
| Col1a1 | CCTCAGGGTATTGCTGGACAAC | ACCACTTGATCCAGAAGGACCTT |
| Col6a3 | TCAAGAGCCTGCAGTGGATG | TGGACACTTCTTGTCTATGCAG |
| Mmp13 | GTTTACCTTCGCCTCACTAG | TCTCTCCTTCCCAGGGCAAGCAT |
| Ucp1 | ACTGCCACACCTCCAGTCATT | CTTTGCCTCACTCAGGATTGG |
| Dio2 | AATTATGCCTCGGAGAAGACCG | GGCAGTTGCCTAGTGAAAGGT |
| Pgc1-α | CCCTGCCATTGTTAAGACC | TGCTGCTGTTCCTGTTTTC |
| Prdm16 | CAGCACGGTGAAGCCATTC | GCGTGCATCCGCTTGTG |
| Ppar-α | TGACAACCAGACGGCTTGTG | GCAGGCGAGGAAAAGATAGTGT |
| Mt-Co1 | CAACGGCGTCGAAGACAAA | TGACGGTCTCCACGGACAT |
| Cox7a1 | CAGCGTCATGGTCAGTCTGT | AGAAAACCGTGTGGCAGAGA |
| Cox8b | GAACCATGAAGCCAACGACT | GCGAAGTTCACAGTGGTTCC |
| Cebpa | GAGCCGAGATAAAGCCAAACA | GCGCAGGCGGTCATTG |
| Cpt1a | AGACCGTGAGGAACTCAAACCTAT | TGAAGAGTCGCTCCCACT |
| Fasn | TTCCAAGACGAAAATGATGC | AATTGTGGGATCAGGAGAGC |
| Adipoq | CCTGGCCACTTTCTCCTCATT | AAGAGGAACAGGAGAGCTTGC |
| Acox1 | CTATGGGATCAGCCAGAAAGG | AGTCAAAGGCATCCACCAAAG |
| Occludin | TTGAAAGTCCACCTCCTTACAGA | CCGGATAAAAAGAGTACGCTGG |
| ZO-1 | GCCGCTAAGAGCACAGCAA | TCCCCACTCTGAAAATGAGGA |
| claudin1 | GGGGACAACATCGTGACCG | AGGAGTCGAAGACTTTGCACT |
| claudin3 | ACCAACTGCGTACAAGACGAG | CAGAGCCGCCAACAGGAAA |
| mucin 2 | AGGGCTCGGAACTCCAGAAA | CCAGGGAATCGGTAGACATCG |
| Reg3γ | ATGCTTCCCCGTATAACCATCA | GGCCATATCTGCATCATACCAG |
| IFN-γ | CCACGGCACAGTCATTGAAAG | TGCTGATGGCCTGATTGTCTT |
| Igrm1 | CTTTCCCAATGTGGTGCTGTG | AACCTCTTTCCCATGCTCTGG |
| Lyz1 | GCCAAGGTCTACAATCGTTGTGAGTTG | CAGTCAGCCAGCTTGACACCACG |
| Defcr1 | TCAAGAGGCTGCAAAGGAAGAGAAC | TGGTCTCCATGTTCAGCGACAGC |
| Defa-rs1 | CACCACCCAAGCTCCAAATACACAG | ATCGTGAGGACCAAAAGCAAATGG |
| Defa21 | CCAGGGGAAGATGACCAGGCTG | TGCAGCGACGATTTCTACAAAGGC |
| CR2 | CCAGGCTGATCCTATCCAAA | GTCCCATTCATGCGTTCTCT |
| fldC | TCCAGAAGCGAACACACCAA | AGTACATGTGGAATGCCTGCT |
| 16S | CGGTGAATACGTTCYCGG | GGWTACCTTGTTACGACTT |

**Detailed Methods**

**IPA treatment**

The IPA (Sigma-Aldrich, 220027) was diluted in sterile PBS to a concentration of 2.5 mg/mL. Mice were orally gavaged with a daily dose of 20 mg/kg.

**Non-targeted metabolomics analysis**

After removing high-molecular-weight substances by pre-cooling methanol precipitation, the supernatant was collected after centrifugation and vacuum-concentrated to dryness. The samples were fully dissolved for injection. The high-performance liquid chromatography system used was a Dionex U3000 system, coupled with a Q Exactive mass spectrometer. For positive ion detection: Buffer A = 99.9% water, 0.1% formic acid; Buffer B = 99.9% acetonitrile, 0.1% formic acid. For negative ion detection: Buffer A = 10 mM ammonium acetate aqueous solution, 5% acetonitrile, pH adjusted to around 9 with ammonia solution; Buffer B = 90% acetonitrile, 10% water, 10 mM ammonium acetate, pH adjusted to around 9 with ammonia solution. The full scan range was set from m/z 70 to 1050. In the full scan, the top 15 ions with the highest intensity were selected for secondary mass spectrometry identification. HCD fragmentation was used for the secondary mass spectrometry sequencing of the parent ions. Data extraction and processing were performed using Compound Discover software, and compound identification was completed through online searches using mzCloud and comprehensive databases like ChemSpider. Metabolites with VIP > 1 and fold change > 2 or < 0.5 (adj. *P*-value < 0.05) were considered as significantly changed metabolites.

**Preparation of serum samples for LC-MS analysis**

Whole blood samples were collected from mice through retro-orbital bleeding and transferred into sterile EP tubes, which were then left at room temperature for one hour. The blood was centrifuged at 4 °C and 5,000 rpm for 10 minutes, and the clear supernatant was aspirated and frozen at -80 °C. For the extraction of polar metabolites from serum for LC-MS analysis, 200 μL of ice-cold methanol (1:4) was added to 50 μL of serum and vortex-mixed for 1 minute. After incubation at room temperature for 10 minutes, the mixture was stored overnight at -20 °C to enhance protein precipitation. The resulting mixture was then centrifuged at 4 °C and 17,000 g for 15 minutes, and the clear supernatant was transferred to LC-MS vials with micro-insert tubes.

**Targeted measurement of IPA using LC-MS**

To elucidate the chemical structure of the analyte with m/z 190.0931 in the serum, we identified IPA in plasma using high-performance liquid chromatography/high-resolution mass spectrometry (HPLC/HRMS), where its retention time and high-resolution mass spectrum matched those of an authentic standard. The concentrations of IPA in serum and feces were analyzed using a MALDI SYNAPT G2-Si triple quadrupole LC-MS instrument (Waters Corp., MA, USA). Chromatographic separation employed an Acquity 1.7-µm UPLC BEH C18 column with dimensions of 100 mm × 2.1 mm, equipped with an Acquity 1.7-µm UPLC BEH C18 pre-column with dimensions of 50 mm × 2.1 mm. The column temperature was maintained at 60 °C. The elution solvent A was a 0.1% formic acid (vol/vol) aqueous solution, while elution solvent B was a 0.1% formic acid (vol/vol) acetonitrile solution. The gradient used was as follows: linear increase from 20% to 25% B (0-5 minutes), followed by an increase to 95% B (0.1 minute), maintained at 95% B (5.1-7 minutes), decreased to 20% B (0.1 minute), and balanced at 20% B (1.9 minutes), with a total run time of 9 minutes and a flow rate of 600 µL/min. The injection volume for standards and extracts was 5 μL (full loop). Detection was achieved through multiple reaction monitoring (MRM) mode in positive ion mode using electrospray ionization, with protonated precursor ions transitioning at m/z 190.09–172.08 and m/z 190.09–130.07. Source parameters in positive ion mode were set as follows: capillary voltage at 2.7 kV, cone voltage at 40 V. The optimal collision energy was 18 a.u., and the dwell time for each transition was set at 30 ms. Quantification was performed using external standardization (ESTD). The IPA standard was analyzed alongside serum samples using the same triple quadrupole quantification method. A linear standard curve (0.5 ppb-100 ppm, r^2^= 0.994726) was generated based on standard concentrations and total ion intensity for calculating the endogenous concentration of IPA. Dilution of serum during sample preparation was also considered in the quantitative analysis.

**Oral Glucose Tolerance Test (OGTT)**

A 0.4 g/mL glucose solution was prepared and allowed to stabilize overnight at room temperature to achieve a consistent hemiacetal conformation. Mice were fasted overnight, without access to food or water, for 12 hours. Following the fasting period, each mouse received an oral glucose (2 g/kg). Blood glucose levels were measured at multiple time points: 30 minutes before glucose administration (time point -30), immediately at the time of glucose administration (time point 0), and at 15, 30, 60, 90, and 120 minutes post-administration. Blood samples were drawn from the tip of the tail vein and glucose concentrations were determined using a blood glucose meter (China Yuwell Group).

**Insulin tolerance test (ITT)**

Mice were fasted for 4 hours prior to the commencement of the experiment. Subsequently, insulin was administered via intraperitoneal injection at varying doses: CD group (0.75 IU/kg), HFD group (1 IU/kg), and HFD+IPA group (1 IU/kg). Blood glucose levels were measured at 0, 15, 30, 60, 90, and 120 min post-injection, with samples collected from the tail vein.

**Fasting hormones and lipid concentrations**

Serum concentrations of leptin, resistin, adiponectin (ADPN), and glucose-dependent insulinotropic polypeptide (GIP) were assessed utilizing enzyme-linked immunosorbent assay kits obtained from Elabscience Biotechnology Co., Ltd. Measurements of total cholesterol, triglycerides, LDL-C, and HDL-C in serum were performed using assay kits sourced from Nanjing Jiancheng Bioengineering Institute. OD values were recorded using an enzyme-linked immunosorbent assay reader in accordance with the provided manufacturer's guidelines.

**In vivo intestine permeability assay**

Mice were subjected to a 12-hour overnight fasting period with water deprivation. On the following day, FITC-dextran (Sigma) was dissolved in sterile saline (125 g/L) and administered via oral gavage at a dose of 0.5 g/kg. Blood samples were collected from mice at 2 hours and 4 hours, and serum was obtained. In a 96-well plate, samples were placed alongside blank wells containing serum from mice gavaged with an equivalent volume of sterile saline. Measure the fluorescence intensity at 485 nm. Quantitative analysis was conducted by referencing a calibration curve derived from known concentrations of serum FITC-dextran.

**Histological analysis**

Subcutaneous adipose tissue (SAT), mesenteric adipose tissue (MAT), brown adipose tissue (BAT), and jejunal tissue were fixed in 4% paraformaldehyde at room temperature for 24 hours. Subsequently, the samples were dehydrated in 100% ethanol for 24 h and subjected to paraffin embedding. 8 μm paraffin sections were stained with hematoxylin and eosin (H&E). Whole-tissue slide scanning was performed using a digital slide scanner (Pannoramic MIDI, 3DHISTECH, Hungary). The SlideViewer 2.7 software (3DHISTECH, Hungary) was used to calculate the size and distribution of adipocytes in five fields of each sample. White areas in brown adipose tissue corresponding to lipid droplets were quantified in five regions of each sample using SlideViewer 2.7.

**Respiratory metabolism monitoring**

Mice were housed individually in a temperature-controlled metabolic cage (CLAMS-16MR/CIS-16MR, Columbus Instruments) set at 30 °C. Before the experiment, mice were acclimated for 2 days in metabolic cages. During the entire experimental period, continuous monitoring was conducted, including measurements of food intake, oxygen consumption (VO_2_), carbon dioxide production (VCO_2_), respiratory exchange ratio (RER, calculated as VCO_2_/VO_2_), and physical activity levels.

**Real-time quantitative PCR**

Total RNA was isolated from adipose tissue using the TRIzol reagent. The concentration and quality of RNA in each sample were determined by analyzing 1 μL of total RNA on a Nanodrop One Bioanalyzer. For subsequent qPCR analysis, 1 µg of total RNA was converted into cDNA using the PrimeScript™ RT Master Mix. Real-time PCR was then performed on the LightCycler 480 II system with TB Green^®^ Premix Ex Taq™ II reagent. *TBP* was utilized as the reference gene. All reactions were conducted in duplicate on a 96-well plate, and the 2^-ΔΔCt^ method was used for data analysis. The specificity and purity of the PCR products were confirmed by analyzing the melt curves generated at the end of the amplification process. Primer sequences for the target genes are detailed in Supplementary Table S3.

**RNAseq**

The RNA integrity was assessed using the RNA Nano 6000 Assay Kit on the Bioanalyzer 2100 system (Agilent Technologies, CA, USA). Index-coded samples were clustered with the TruSeq PE Cluster Kit v3-cBot-HS (Illumina) on a cBot Cluster Generation System, following the manufacturer's instructions. Subsequently, the prepared libraries were subjected to sequencing on an Illumina Novaseq platform, generating 150 bp paired-end reads. Read counting mapped to individual genes was performed using FeatureCounts v1.5.0-p3, and the Fragments Per Kilobase Million (FPKM) for each gene were calculated based on gene length and mapped reads count. The analysis of differential expression between two conditions/groups, each with two biological replicates, was conducted using the DESeq2 R package (1.20.0).

**16S rRNA gene sequencing**

Bacterial DNA extraction from fecal samples was conducted using the DNeasy PowerSoil kit (Qiagen, Hilden, Germany). The DNA concentration and integrity were evaluated using a NanoDrop 2000 spectrophotometer (Thermo Fisher Scientific, USA) and agarose gel electrophoresis, respectively. PCR amplification targeting the V3-V4 hypervariable regions of the bacterial 16S rRNA gene was performed in 25 μl reactions with universal primer pairs (343F: 5′-TACGGRAGGCAGCAG-3′; 798R: 5′-AGGGTATCTAATCCT-3′). The reverse primer included a sample barcode, and both primers were linked with an Illumina sequencing adapter. Amplicon quality was verified through gel electrophoresis. Purification of PCR products was accomplished using Agencourt AMPure XP beads, and quantification was conducted using the Qubit dsDNA assay kit. Subsequently, concentrations were adjusted for sequencing. The sequencing process was carried out on an Illumina NovaSeq6000 platform with two paired-end read cycles of 250 bases each.

**Western blot**

Proteins were extracted using RIPA lysis buffer (Thermo Scientific) containing phosphatase inhibitors, while maintaining the samples on ice. Following separation through 10% SDS-PAGE gel electrophoresis, the proteins were transferred onto a NC membrane. The membrane was then blocked with 5% BSA at room temperature for 2 h, followed by an overnight incubation with the primary antibody at 4 ℃. Subsequently, an HRP-conjugated secondary antibody was applied and incubated at room temperature for 2 h. The signal was detected using the ECL chemiluminescence substrate kit (Thermo Scientific).

**Immunofluorescence**

Mouse ileum paraffin sections were prepared and stored at -20 °C until use. Prior to use, the sections were deparaffinized in xylene and subjected to antigen retrieval. They were then incubated at room temperature for 1 hour in a blocking buffer containing 0.3% Triton X-100 and 5% goat serum in PBS. Primary antibodies were applied and incubated overnight at 4 °C. Secondary antibodies were incubated for 2 hours at room temperature in the dark. DAPI staining was performed for 5 minutes at room temperature. The primary antibodies used were E-Cadherin mouse antibody (CST) and Lysozyme rabbit antibody (CST). The fluorescent secondary antibodies used were Alexa Fluor 488 goat anti-rabbit IgG (Invitrogen) and Alexa Fluor 568 goat anti-mouse IgG (Invitrogen). Images were acquired using a confocal microscope (Carl Zeiss LSM880).

**Statistics**

Normality was evaluated using the Shapiro-Wilk test. For data that follows a normal distribution, two-tailed Student's *t*-test was employed to determine statistical differences between the two groups. For comparisons involving multiple groups, either one-way or two-way analysis of variance (ANOVA) was performed, followed by Bonferroni's multiple comparison test. In cases of non-normally distributed data, the Wilcoxon test or Mann-Whitney *U* test was applied for assessing differences between two groups, and the Kruskal-Wallis test or Dunn's multiple comparison test for multiple groups. All data were presented as mean ± SEM, and categorical variables were expressed as percentages (%). Categorical variable comparisons utilized Fisher's exact test or χ2 test, while Pearson correlation analysis was employed for assessing correlations. *P*＜0.05 was considered significant in all analyses (* denotes *P*＜0.05, ***P*＜0.01, ****P*＜0.001). GraphPad Prism 8.0 and OriginPro 2023 were used for generating charts and statistical data.
